# Greater male than female variability in regional brain structure across the lifespan

**DOI:** 10.1002/hbm.25204

**Published:** 2020-10-12

**Authors:** Lara M Wierenga, Gaelle E Doucet, Danai Dima, Ingrid Agartz, Moji Aghajani, Theophilus N Akudjedu, Anton Albajes‐Eizagirre, Dag Alnæs, Kathryn I Alpert, Ole A Andreassen, Alan Anticevic, Philip Asherson, Tobias Banaschewski, Nuria Bargallo, Sarah Baumeister, Ramona Baur‐Streubel, Alessandro Bertolino, Aurora Bonvino, Dorret I Boomsma, Stefan Borgwardt, Josiane Bourque, Anouk den Braber, Daniel Brandeis, Alan Breier, Henry Brodaty, Rachel M Brouwer, Jan K Buitelaar, Geraldo F Busatto, Vince D Calhoun, Erick J Canales‐Rodríguez, Dara M Cannon, Xavier Caseras, Francisco X Castellanos, Tiffany M Chaim‐Avancini, Christopher RK Ching, Vincent P Clark, Patricia J Conrod, Annette Conzelmann, Fabrice Crivello, Christopher G Davey, Erin W Dickie, Stefan Ehrlich, Dennis van't Ent, Simon E Fisher, Jean‐Paul Fouche, Barbara Franke, Paola Fuentes‐Claramonte, Eco JC de Geus, Annabella Di Giorgio, David C Glahn, Ian H Gotlib, Hans J Grabe, Oliver Gruber, Patricia Gruner, Raquel E Gur, Ruben C Gur, Tiril P Gurholt, Lieuwe de Haan, Beathe Haatveit, Ben J Harrison, Catharina A Hartman, Sean N Hatton, Dirk J Heslenfeld, Odile A van den Heuvel, Ian B Hickie, Pieter J Hoekstra, Sarah Hohmann, Avram J Holmes, Martine Hoogman, Norbert Hosten, Fleur M Howells, Hilleke E Hulshoff Pol, Chaim Huyser, Neda Jahanshad, Anthony C James, Jiyang Jiang, Erik G Jönsson, John A Joska, Andrew J Kalnin, Karolinska Schizophrenia Project (KaSP) Consortium, Marieke Klein, Laura Koenders, Knut K Kolskår, Bernd Krämer, Jonna Kuntsi, Jim Lagopoulos, Luisa Lazaro, Irina S Lebedeva, Phil H Lee, Christine Lochner, Marise WJ Machielsen, Sophie Maingault, Nicholas G Martin, Ignacio Martínez‐Zalacaín, David Mataix‐Cols, Bernard Mazoyer, Brenna C McDonald, Colm McDonald, Andrew M McIntosh, Katie L McMahon, Genevieve McPhilemy, Dennis van der Meer, José M Menchón, Jilly Naaijen, Lars Nyberg, Jaap Oosterlaan, Yannis Paloyelis, Paul Pauli, Giulio Pergola, Edith Pomarol‐Clotet, Maria J Portella, Joaquim Radua, Andreas Reif, Geneviève Richard, Joshua L Roffman, Pedro GP Rosa, Matthew D Sacchet, Perminder S Sachdev, Raymond Salvador, Salvador Sarró, Theodore D Satterthwaite, Andrew J Saykin, Mauricio H Serpa, Kang Sim, Andrew Simmons, Jordan W Smoller, Iris E Sommer, Carles Soriano‐Mas, Dan J Stein, Lachlan T Strike, Philip R Szeszko, Henk S Temmingh, Sophia I Thomopoulos, Alexander S Tomyshev, Julian N Trollor, Anne Uhlmann, Ilya M Veer, Dick J Veltman, Aristotle Voineskos, Henry Völzke, Henrik Walter, Lei Wang, Yang Wang, Bernd Weber, Wei Wen, John D West, Lars T Westlye, Heather C Whalley, Steven CR Williams, Katharina Wittfeld, Daniel H Wolf, Margaret J Wright, Yuliya N Yoncheva, Marcus V Zanetti, Georg C Ziegler, Greig I de Zubicaray, Paul M Thompson, Eveline A Crone, Sophia Frangou, Christian K Tamnes

**Affiliations:** ^1^ Institute of Psychology Leiden University Leiden The Netherlands; ^2^ Leiden Institute for Brain and Cognition Leiden The Netherlands; ^3^ Department of Psychiatry Icahn School of Medicine at Mount Sinai New York New York USA; ^4^ Boys Town National Research Hospital Omaha Nebraska USA; ^5^ Department of Psychology, School of Arts and Social Sciences, City University of London London UK; ^6^ Department of Neuroimaging, Institute of Psychiatry, Psychology and Neuroscience King's College London London UK; ^7^ Norwegian Centre for Mental Disorders Research (NORMENT), Division of Mental Health and Addiction, Institute of Clinical Medicine University of Oslo Oslo Norway; ^8^ Department of Psychiatric Research Diakonhjemmet Hospital Oslo Norway; ^9^ Centre for Psychiatry Research, Department of Clinical Neuroscience, Karolinska Institutet, & Stockholm Health Care Services Stockholm County Council Stockholm Sweden; ^10^ Department of Psychiatry, Amsterdam Neuroscience, Amsterdam UMC Vrije Universiteit Amsterdam The Netherlands; ^11^ Department of Research & Innovation GGZ inGeest Amsterdam The Netherlands; ^12^ Institute of Education and Child Studies, Forensic Family and Youth Care Leiden University Leiden The Netherlands; ^13^ Centre for Neuroimaging & Cognitive Genomics (NICOG), Clinical Neuroimaging Laboratory, NCBES Galway Neuroscience Centre, College of Medicine Nursing and Health Sciences National University of Ireland Galway Galway Ireland; ^14^ Institute of Medical Imaging & Visualisation, Faculty of Health & Social Sciences Bournemouth University Bournemouth UK; ^15^ FIDMAG Germanes Hospitalàries Research Foundation Barcelona Spain; ^16^ Centro de Investigación Biomédica en Red de Salud Mental (CIBERSAM) Madrid Spain; ^17^ Institut d'Investigacions Biomèdiques August Pi i Sunyer (IDIBAPS) Barcelona Spain; ^18^ Norwegian Centre for Mental Disorders Research (NORMENT), Division of Mental Health and Addiction Oslo University Hospital Oslo Norway; ^19^ Department of Psychiatry and Behavioral Sciences Northwestern University Feinberg School of Medicine Chicago Illinois USA; ^20^ Department of Psychiatry Yale University New Haven Connecticut USA; ^21^ Social, Genetic and Developmental Psychiatry Centre, Institute of Psychiatry, Psychology and Neuroscience King's College London London UK; ^22^ Department of Child and Adolescent Psychiatry and Psychotherapy, Central Institute of Mental Health University of Heidelberg, Medical Faculty Mannheim Mannheim Germany; ^23^ Imaging Diagnostic Center Hospital Clínic Barcelona Spain; ^24^ Magnetic Resonance Image Core Facility IDIBAPS Barcelona Spain; ^25^ Department for Clinical Psychology Würzburg University Margetshöchheim Germany; ^26^ Department of Basic Medical Science, Neuroscience and Sense Organs University of Bari Aldo Moro Bari Italy; ^27^ Department of Biological Psychology VU University Amsterdam Amsterdam The Netherlands; ^28^ Department of Psychiatry University of Basel Basel Switzerland; ^29^ Department of Psychiatry University of Lübeck Lübeck Germany; ^30^ Department of Psychiatry University of Pennsylvania Philadelphia Pennsylvania USA; ^31^ CHU Sainte‐Justine Research Center Montreal Quebec Canada; ^32^ Alzheimer Center Amsterdam UMC, Location VUMC Amsterdam The Netherlands; ^33^ Department of Child and Adolescent Psychiatry and Psychotherapy, Psychiatric Hospital University of Zurich Zurich Switzerland; ^34^ Zurich Center for Integrative Human Physiology University of Zurich Zurich Switzerland; ^35^ Neuroscience Centre Zurich University and ETH Zurich Zurich Switzerland; ^36^ Department of Psychiatry Indiana University School of Medicine Indianapolis Indiana USA; ^37^ Centre for Healthy Brain Ageing, School of Psychiatry University of New South Wales Sydney New South Wales Australia; ^38^ Dementia Centre for Research Collaboration, School of Psychiatry University of New South Wales Sydney New South Wales Australia; ^39^ Department of Psychiatry, University Medical Center Utrecht Brain Center Utrecht University Utrecht The Netherlands; ^40^ Department of Cognitive Neuroscience Radboud University Medical Centre Nijmegen The Netherlands; ^41^ Karakter Child and Adolescent Psychiatry University Centre Nijmegen The Netherlands; ^42^ Laboratory of Psychiatric Neuroimaging (LIM‐21), Departamento e Instituto de Psiquiatria, Hospital das Clinicas HCFMUSP, Faculdade de Medicina Universidade de São Paulo São Paulo Brazil; ^43^ Tri‐institutional Center for Translational Research in Neuroimaging and Data Science (TReNDS) Georgia State, Georgia Tech Atlanta Georgia USA; ^44^ MRC Centre for Neuropsychiatric Genetics and Genomics Cardiff University Cardiff UK; ^45^ Department of Child and Adolescent Psychiatry NYU Grossman School of Medicine New York New York USA; ^46^ Nathan Kline Institute for Psychiatric Research Orangeburg New York USA; ^47^ Imaging Genetics Center, Mark and Mary Stevens Neuroimaging and Informatics Institute, Keck School of Medicine University of Southern California Los Angeles California USA; ^48^ Psychology Clinical Neuroscience Center, Department of Psychology University of New Mexico Albuquerque New Mexico USA; ^49^ Mind Research Network Albuquerque New Mexico USA; ^50^ Department of Psychiatry University of Montreal Montreal Canada; ^51^ Department of Child and Adolescent Psychiatry, Psychosomatics and Psychotherapy University of Tübingen Tübingen Germany; ^52^ Department of Psychology (Clinical Psychology II) PFH – Private University of Applied Sciences Göttingen Germany; ^53^ Groupe d'Imagerie Neurofonctionnelle Institut des Maladies Neurodégénératives Bordeaux France; ^54^ Centre for Youth Mental Health University of Melbourne Parkville Victoria Australia; ^55^ Orygen Parkville Victoria Australia; ^56^ Campbell Family Mental Health Institute, Centre for Addiction and Mental Health, Department of Psychiatry University of Toronto Toronto Canada; ^57^ Department of Psychiatry University of Toronto Toronto Ontario Canada; ^58^ Division of Psychological & Social Medicine and Developmental Neurosciences; Technische Universität Dresden, Faculty of Medicine University Hospital C.G. Carus Dresden Germany; ^59^ Language and Genetics Department Max Planck Institute for Psycholinguistics Nijmegen The Netherlands; ^60^ Donders Institute for Brain, Cognition and Behaviour Radboud University Nijmegen The Netherlands; ^61^ Department of Psychiatry and Neuroscience Institute University of Cape Town Cape Town Western Cape South Africa; ^62^ Department of Human Genetics Radboud University Medical Center Nijmegen The Netherlands; ^63^ Department of Psychiatry Radboud University Medical Center Nijmegen The Netherlands; ^64^ IRCCS Casa Sollievo della Sofferenza San Giovanni Rotondo Italy; ^65^ Tommy Fuss Center for Neuropsychiatric Disease Research, Department of Psychiatry Boston Children's Hospital and Harvard Medical School Boston Massachusetts USA; ^66^ Olin Center for Neuropsychiatric Research, Institute of Living Hartford Hospital Hartford Connecticut USA; ^67^ Department of Psychology Stanford University Stanford California USA; ^68^ Department of Psychiatry and Psychotherapy University Medicine Greifswald Greifswald Germany; ^69^ German Center for Neurodegenerative Diseases (DZNE) Site Rostock/Greifswald Greifswald Germany; ^70^ Section for Experimental Psychopathology and Neuroimaging, Department of General Psychiatry Heidelberg University Hospital Heidelberg Germany; ^71^ Lifespan Brain Institute Children's Hospital of Philadelphia Philadelphia Pennsylvania USA; ^72^ Department of Early Psychosis Amsterdam UMC Amsterdam The Netherlands; ^73^ Melbourne Neuropsychiatry Centre, Department of Psychiatry The University of Melbourne & Melbourne Health Melbourne Australia; ^74^ Interdisciplinary Center Psychopathology and Emotion regulation University of Groningen, University Medical Center Groningen Groningen The Netherlands; ^75^ Brain and Mind Centre University of Sydney Sydney New South Wales Australia; ^76^ Department of Neurosciences University of California San Diego La Jolla California USA; ^77^ Departments of Experimental and Clinical Psychology Vrije Universiteit Amsterdam Amsterdam The Netherlands; ^78^ Department of Anatomy & Neurosciences, Amsterdam Neuroscience Amsterdam UMC, Vrije Universiteit Amsterdam Amsterdam The Netherlands; ^79^ Department of Psychiatry University of Groningen, University Medical Center Groningen Groningen The Netherlands; ^80^ Department of Psychology Yale University New Haven Connecticut USA; ^81^ Department of Psychiatry Massachusetts General Hospital Boston Massachusetts USA; ^82^ Institute of Diagnostic Radiology and Neuroradiology University Medicine Greifswald Greifswald Germany; ^83^ Neuroscience Institute University of Cape Town Cape Town Western Cape South Africa; ^84^ Department of Psychiatry and Mental Health University of Cape Town Cape Town Western Cape South Africa; ^85^ De Bascule, Academic center child and adolescent psychiatry Duivendrecht The Netherlands; ^86^ Amsterdam UMC Department of Child and Adolescent Psychiatry Amsterdam The Netherlands; ^87^ Department of Psychiatry Warneford Hospital Oxford UK; ^88^ Highfield Unit Warneford Hospital Oxford UK; ^89^ Department of Radiology The Ohio State University College of Medicine Columbus Ohio USA; ^90^ Department of Psychology University of Oslo Oslo Norway; ^91^ Sunnaas Rehabilitation Hospital HT Nesodden Norway; ^92^ Sunshine Coast Mind and Neuroscience Thompson Institute Birtinya Queensland Australia; ^93^ University of the Sunshine Coast Sunshine Coast Queensland Australia; ^94^ Department of Child and Adolescent Psychiatry and Psychology Hospital Clínic Barcelona Spain; ^95^ August Pi i Sunyer Biomedical Research Institut (IDIBAPS) Barcelona Spain; ^96^ Department of Medicine University of Barcelona Barcelona Spain; ^97^ Laboratory of Neuroimaging and Multimodal Analysis Mental Health Research Center Moscow Russia; ^98^ Department of Psychiatry Harvard Medical School Boston Massachusetts USA; ^99^ SA MRC Unit on Risk and Resilience in Mental Disorders, Department of Psychiatry Stellenbosch University Cape Town Western Cape South Africa; ^100^ Department of Psychiatry Academic Medical Center Amsterdam The Netherlands; ^101^ Institut des maladies neurodégénératives Université de Bordeaux Bordeaux France; ^102^ Genetic Epidemiology QIMR Berghofer Medical Research Institute Brisbane Queensland Australia; ^103^ Department of Psychiatry, Bellvitge University Hospital Bellvitge Biomedical Research Institute‐IDIBELL Barcelona Spain; ^104^ Department of Clinical Sciences University of Barcelona Barcelona Spain; ^105^ University of Bordeaux Bordeaux France; ^106^ Bordeaux University Hospital Bordeaux France; ^107^ Department of Radiology and Imaging Sciences Indiana University School of Medicine Indianapolis Indiana USA; ^108^ Division of Psychiatry University of Edinburgh Edinburgh UK; ^109^ Herston Imaging Research Facility and School of Clinical Sciences Queensland University of Technology (QUT) Brisbane Queensland Australia; ^110^ Faculty of Health, Institute of Health and Biomedical Innovation Queensland University of Technology (QUT) Brisbane Queensland Australia; ^111^ School of Mental Health and Neuroscience, Faculty of Health, Medicine and Life Sciences Maastricht University Maastricht The Netherlands; ^112^ Department of Radiation Sciences Umeå University Umeå Sweden; ^113^ Department of Integrative Medical Biology Umeå University Umeå Sweden; ^114^ Emma Children's Hospital, Amsterdam UMC University of Amsterdam and Vrije Universiteit Amsterdam Emma Neuroscience Group, Department of Pediatrics, Amsterdam Reproduction & Development Amsterdam The Netherlands; ^115^ Clinical Neuropsychology Section Vrije Universiteit Amsterdam Amsterdam The Netherlands; ^116^ Department of Psychology University of Würzburg Würzburg Germany; ^117^ Centre of Mental Health, Medical Faculty University of Würzburg Würzburg Germany; ^118^ Lieber Institute for Brain Development Johns Hopkins Medical Campus Baltimore Mary Land USA; ^119^ Department of Psychiatry Institut d'Investigació Biomèdica Sant Pau Barcelona Spain; ^120^ Early Psychosis: Interventions and Clinical‐detection (EPIC) lab, Department of Psychosis Studies Institute of Psychiatry, Psychology and Neuroscience, King's College London London UK; ^121^ Department of Psychiatry, Psychosomatic Medicine and Psychotherapy University Hospital Frankfurt Frankfur am Maint Germany; ^122^ Department of Psychiatry Massachusetts General Hospital and Harvard Medical School Charlestown Massachusetts USA; ^123^ Center for Depression, Anxiety, and Stress Research McLean Hospital, Harvard Medical School Belmont Massachusetts USA; ^124^ Neuropsychiatric Institute The Prince of Wales Hospital Randwick New South Wales Australia; ^125^ Indiana Alzheimer Disease Center Indianapolis Indiana USA; ^126^ West Region, Institute of Mental Health Singapore Singapore; ^127^ Yong Loo Lin School of Medicine National University of Singapore Singapore; ^128^ Department of Neuroimaging, Institute of Psychiatry Psychology and Neurology, King's College London London UK; ^129^ Psychiatric and Neurodevelopmental Genetics Unit, Center for Genomic Medicine Massachusetts General Hospital Boston Massachusetts USA; ^130^ Department of Biomedical Sciences of Cells and Systems, Rijksuniversiteit Groningen University Medical Center Groningen Groningen The Netherlands; ^131^ Department of Psychobiology and Methodology in Health Sciences Universitat Autònoma de Barcelona Barcelona Spain; ^132^ SAMRC Unit on Risk & Resilience in Mental Disorders, Dept of Psychiatry & Neuroscience Institute University of Cape Town Cape Town Western Cape South Africa; ^133^ Queensland Brain Institute University of Queensland Brisbane Queensland Australia; ^134^ Mental Illness Research, Education and Clinical Center (MIRECC) James J. Peters VA Medical Center New York New York USA; ^135^ Department of Child and Adolescent Psychiatry and Psychotherapy Faculty of Medicine Carl Gustav Carus of TU Dresden Dresden Germany; ^136^ Department of Psychiatry and Psychotherapy CCM, Charité ‐ Universitätsmedizin Berlin, corporate member of Freie Universität Berlin Humboldt‐Universität zu Berlin, and Berlin Institute of Health Berlin Germany; ^137^ Department of Psychiatry & Amsterdam Neuroscience Amsterdam UMC, location VUMC Amsterdam The Netherlands; ^138^ Institute for Community Medicine University Medicine Greifswald Greifswald Germany; ^139^ DZHK (German Centre for Cardiovascular Research), partner site Greifswald Greifswald Germany; ^140^ DZD (German Center for Diabetes Research), partner site Greifswald Greifswald Germany; ^141^ Department of Radiology Medical College of Wisconsin Milwaukee Wisconsin USA; ^142^ Institute for Experimental Epileptology and Cognition Research University Hospital Bonn Bonn Germany; ^143^ Division of Psychiatry Royal Edinburgh Hospital Edinburgh UK; ^144^ Department of Neuroimaging King's College London London UK; ^145^ Centre for Advanced Imaging University of Queensland Brisbane Queensland Australia; ^146^ Department of Child and Adolescent Psychiatry, NYU Child Study Center Hassenfeld Children's Hospital at NYU Langone New York New York USA; ^147^ Instituto de Ensino e Pesquisa Hospital Sírio‐Libanês São Paulo Brazil; ^148^ Division of Molecular Psychiatry, Center of Mental Health University of Würzburg Würzburg Germany; ^149^ Department of Psychology, Education and Child Studies (DPECS), Erasmus School of Social and Behavioral Sciences Erasmus University Rotterdam The Netherlands; ^150^ Centre for Brain Health University of British Columbia Vancouver British Columbia Canada; ^151^ PROMENTA Research Center, Department of Psychology University of Oslo Oslo Norway

## Abstract

For many traits, males show greater variability than females, with possible implications for understanding sex differences in health and disease. Here, the ENIGMA (Enhancing Neuro Imaging Genetics through Meta‐Analysis) Consortium presents the largest‐ever mega‐analysis of sex differences in variability of brain structure, based on international data spanning nine decades of life. Subcortical volumes, cortical surface area and cortical thickness were assessed in MRI data of 16,683 healthy individuals 1‐90 years old (47% females). We observed significant patterns of greater male than female between‐subject variance for all subcortical volumetric measures, all cortical surface area measures, and 60% of cortical thickness measures. This pattern was stable across the lifespan for 50% of the subcortical structures, 70% of the regional area measures, and nearly all regions for thickness. Our findings that these sex differences are present in childhood implicate early life genetic or gene‐environment interaction mechanisms. The findings highlight the importance of individual differences within the sexes, that may underpin sex‐specific vulnerability to disorders.

## Graphical abstract

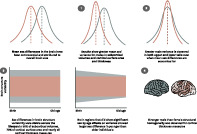

## 1 INTRODUCTION

For a diverse set of human traits and behaviors, males are often reported to show greater variability than females (Hyde 2014). This sex difference has been noted for aspects of personality (Borkenau, McCrae, and Terracciano 2013), cognitive abilities (Arden and Plomin 2006; Johnson, Carothers, and Deary 2008; Roalf et al. 2014), and school achievement (Baye and Monseur 2016). A fundamental question is to what degree these sex differences are related to genetic mechanisms or social factors, or their interactions. Lehre et al. (2009) found compelling evidence for an early genetic or in utero contribution, reporting greater male variability in anthropometric traits (e.g. body weight and height, blood parameters) already detectable at birth. Recent studies suggest greater male variability also in brain structure and its development (Forde et al. 2020; Ritchie et al. 2018; Wierenga et al. 2018, 2019), but studies with larger samples that cover both early childhood and old age are critically needed. Specifically, we do not know when sex differences in variability in brain structure emerge and whether they change with development and throughout life. Yet, data on this could inform us on the origins and factors that influence this phenomenon. For this reason, we set out to analyze magnetic resonance imaging (MRI) data from a large sample of individuals across a very wide age range (n = 16,683, age 1‐90) to robustly characterize sex differences in variability of brain structure and test how these differences interact with age.

Many prior studies report sex differences in brain structure, but the specificity, regional pattern and functional relevance of such effects are not clear (Herting et al. 2018; Koolschijn and Crone 2013; Marwha, Halari, and Eliot 2017; Ruigrok et al. 2014; Tan et al. 2016). One reason could be that most studies have examined mean differences between the sexes, while sex differences in variability remain understudied (Del Giudice et al. 2016; Joel et al. 2015). As mean and variance measure two different aspects of the distribution (center and spread), knowledge on variance effects may provide important insights into sex differences in the brain. Recent studies observed greater male variance for subcortical volumes and for cortical surface area to a larger extent than for cortical thickness (Ritchie et al. 2018; Wierenga et al. 2018, 2019). However, further studies are needed to explore regional patterns of variance differences, and, critically, to test how sex differences in variability in the brain unfold across the lifespan.

An important question pertains to the mechanisms involved in sex differences in variability. It is hypothesized that the lack of two parental X‐chromosomal copies in human males may directly relate to greater variability and vulnerability to developmental disorders in males compared to females (Arnold 2012). All cells in males express an X‐linked variant, while female brain tissues show two variants. In females, one of the X‐chromosomes is randomly silenced, as such neighboring cells may have different X related genetic expression (Wu et al. 2014). Consequently, one could expect that in addition to greater variability across the population, interregional anatomical correlations may be stronger in male relative to female brains. This was indeed observed for a number of regional brain volumes in children and adolescents, showing greater within‐subject homogeneity across regions in males than females (Wierenga et al. 2018). These results remain to be replicated in larger samples as they may provide clues about mechanisms and risk factors in neurodevelopmental disorders (e.g. attention‐deficit/hyperactivity disorder and autism spectrum disorder) that show sex differences in prevalence (Bao and Swaab 2010), age of onset, heritability rates (Costello et al. 2003), or severity of symptoms and course (Goldstein, Seidman, and O'brien 2002).

In the present study, we performed mega‐analyses on data from the enhancing neuroimaging genetics through meta‐analysis (ENIGMA) Lifespan working group (Dima et al., 2020; Frangou et al., 2020; Jahanshad and Thompson 2016). A mega‐analysis allows for analyses of data from multiple sites with a single statistical model that fits all data and simultaneously accounting for the effect of site. Successfully pooling lifespan data was recently shown in a study combining 18 datasets to derive age trends of brain structure (Pomponio et al. 2020). This contrasts with meta‐analysis where summary statistics are combined and weighted from data that is analyzed at each site (van Erp et al. 2019). MRI data from a large sample (*n =* 16,683) of participants aged 1 to 90 years was included. We investigated subcortical volumes and regional cortical surface area and thickness. Our first aim was to replicate previous findings of greater male variability in brain structure in a substantially larger sample. Based on prior studies (Forde et al. 2020; Ritchie et al. 2018; Wierenga et al. 2018, 2019) and reports of somewhat greater genetic effect on surface area than thickness (Eyler et al. 2011; Kremen et al. 2013), we hypothesized that greater male variance would be more pronounced for subcortical volumes and cortical surface area than for cortical thickness, and that greater male variance would be observed at both upper and lower ends of the distribution. Our second aim was to test whether observed sex differences in variability of brain structure are stable across the lifespan from birth until 90 years of age, or e.g. increase with the accumulation of experiences (Pfefferbaum, Sullivan, and Carmelli 2004). Third, in line with the single X‐chromosome hypothesis, we aimed to replicate whether males show greater interregional anatomical correlations (i.e. within‐subject homogeneity) across brain regions that show greater male compared to female variance (Wierenga et al. 2019).

## 2 METHODS

### 2.1 Participants

The datasets analyzed in the present study were from the Lifespan working group within the ENIGMA Consortium (Jahanshad and Thompson 2016). There were 78 independent samples with MRI data, in total including 16,683 (7,966 males) healthy participants aged 1‐90 years from diverse ethnic backgrounds (see detailed descriptions at the cohort level in Table 1). Samples were drawn from the general population or were healthy controls in clinical studies. Screening procedures and the eligibility criteria (e.g. head trauma, neurological history) may be found in Supplemental Table 1. Participants in each cohort gave written informed consent at the local sites. Furthermore, at each site local research ethics committees or Institutional Review Boards gave approval for the data collection, and all local institutional review boards permitted the use of extracted measures of the completely anonymized data that were used in the present study.

**TABLE 1:** Sex distributions and age of subjects by sample

Sample	Total *N*	Sex	*N*	Age		
				Mean	*SD*	Range
EDINBURGH	55	Male	20	23.9	2.5	18.5–28.4
		Female	35	23.7	3.1	18.6–30.6
UNIBA	131	Male	67	30.3	10.0	18.0–63.0
		Female	64	24.3	6.8	18.0–52.0
Tuebingen	50	Male	22	38.4	11.1	26.0–61.0
		Female	28	42.2	12.5	24.0–61.0
GSP	2009	Male	894	27.8	16.8	18.0–90.0
		Female	1115	26.7	16.2	18.0–89.0
Melbourne	102	Male	54	19.5	2.9	15.0–25.0
		Female	48	19.6	3.1	15.0–26.0
HMS	55	Male	21	41.3	11.2	24.0–59.0
		Female	34	38.5	12.8	19.0–64.0
ENIGMA‐OCD (1)	66	Male	30	30.6	8.9	19.0–56.0
		Female	36	35.1	10.9	18.0–61.0
NUIG	93	Male	54	34.1	11.6	18.0–57.0
		Female	39	39.0	11.0	18.0–58.0
NeuroIMAGE	383	Male	177	16.8	3.6	7.7–28.5
		Female	206	17.0	3.8	7.8–28.6
CAMH	141	Male	72	43.2	18.9	18.0–86.0
		Female	69	44.1	19.8	18.0–82.0
Basel	44	Male	17	25.7	4.5	19.0–35.0
		Female	27	25.3	4.2	19.0–39.0
Bordeaux	452	Male	220	26.9	7.8	18.0–57.0
		Female	232	26.6	7.7	18.0–56.0
FBIRN	174	Male	124	37.6	11.3	19.0–60.0
		Female	50	37.4	11.3	19.0–58.0
KaSP	32	Male	15	27.4	5.5	21.0–43.0
		Female	17	27.6	5.9	20.0–37.0
CODE	72	Male	31	43.7	12.4	25.0–64.0
		Female	41	36.6	13.4	20.0–63.0
Indiana (1)	49	Male	9	71.9	6.6	63.0–80.0
		Female	40	60.4	11.6	37.0–84.0
COMPULS/TS EUROTRAIN	53	Male	36	10.8	1.0	8.7–12.9
		Female	17	11.0	1.1	9.2–12.9
FIDMAG	123	Male	54	36.4	8.5	19.0–63.0
		Female	69	38.4	11.2	19.0–64.0
NU	79	Male	46	31.6	14.5	14.6–66.3
		Female	33	34.4	15.3	14.2–67.9
SHIP‐TREND	818	Male	467	50.5	14.4	22.0–81.0
		Female	351	49.6	14.0	21.0–81.0
SHIP‐2	373	Male	207	55.6	12.8	31.0–84.0
		Female	166	54.4	12.0	32.0–88.0
QTIM	340	Male	111	22.5	3.3	16.0–29.3
		Female	229	22.7	3.4	16.1–30.0
Betula	287	Male	136	61.6	12.5	25.5–81.3
		Female	151	64.1	13.1	25.7–80.9
TOP	303	Male	159	34.5	8.8	18.3–56.2
		Female	144	36.3	10.9	19.3–73.4
HUBIN	102	Male	69	42.1	9.0	19.4–54.9
		Female	33	41.7	8.5	19.9–56.2
StrokeMRI	52	Male	19	47.9	20.8	20.0–77.0
		Female	33	43.6	23.0	18.0–78.0
AMC	99	Male	65	22.5	3.4	17.0–32.0
		Female	34	23.6	3.3	18.0–29.0
NESDA	65	Male	23	40.7	9.7	23.0–56.0
		Female	42	40.1	9.9	21.0–54.0
Barcelona (1)	30	Male	14	15.1	1.5	13.0–17.0
		Female	16	14.9	2.1	11.0–17.0
Barcelona (2)	44	Male	24	14.4	1.8	11.0–17.0
		Female	20	14.8	2.4	11.0–17.0
Stages‐Dep	32	Male	9	46.6	8.4	37.0–58.0
		Female	23	45.8	8.2	27.0–58.0
IMpACT	144	Male	57	34.2	11.0	19.0–62.0
		Female	87	37.2	12.6	19.0–63.0
BIG	1319	Male	657	29.8	15.4	17.0–82.0
		Female	662	26.9	12.9	13.0–79.0
IMH Stanford	56	Male	22	36.0	10.5	20.4–60.5
	34	Female	34	37.5	10.8	18.9–56.3
MCIC (1) + (2)	93	Male	63	32.8	12.2	18.0–58.0
		Female	30	32.5	11.9	19.0–60.0
OLIN	599	Male	237	36.3	13.3	22.0–86.5
		Female	362	35.9	12.8	21.0–74.0
Neuroventure	137	Male	62	13.7	0.6	12.4–14.9
		Female	75	13.6	0.7	12.3–14.9
CIAM	30	Male	16	27.1	5.9	19.0–40.0
		Female	14	26.1	3.8	20.0–33.0
ENIGMA‐HIV	31	Male	16	25.6	4.7	19.0–33.0
		Female	15	23.9	4.1	20.0–32.0
Meth‐CT	62	Female	13	26.1	4.1	19.0–34.0
		Males	49	27.0	7.9	18.0–53.0
ENIGMA‐OCD	26	Male	10	34.6	13.6	19.0–56.0
		Female	16	28.8	7.8	20.0–46.0
Oxford	38	Male	18	16.5	1.6	14.1–18.9
		Female	20	15.9	1.1	13.7–17.7
Yale	23	Male	12	14.4	2.4	10.3–17.5
		Female	11	14.0	2.0	9.9–16.5
Sao Paulo‐1	69	Male	45	27.1	5.6	18.0–42.0
		Female	24	27.5	6.4	17.0–43.0
Sao Paulo‐3	85	Male	45	28.2	7.3	18.0–43.0
		Female	40	32.7	8.8	18.0–50.0
ENIGMA‐OCD (2)	49	Male	19	32.1	7.8	24.0–53.0
		Female	30	31.3	7.7	21.0–50.0
ENIGMA‐OCD (3)	35	Male	16	42.9	12.9	22.5–64.0
		Female	19	36.0	8.8	21.5–49.3
ENIGMA‐OCD (4)	23	Male	9	13.1	2.9	8.8–15.9
		Female	14	13.8	2.4	8.7–16.8
ENIGMA‐OCD (5)	33	Male	12	30.7	8.8	21.0–53.0
		Female	21	39.2	11.5	24.0–63.0
SYDNEY	157	Male	65	42.0	22.4	12.0–84.0
		Female	92	37.1	21.7	13.0–78.0
IMH	79	Male	50	30.7	8.3	23.0–53.9
		Female	29	34.2	12.4	20.4–59.0
UPENN	187	Male	86	35.7	12.9	18.0–71.0
		Female	101	35.8	14.7	16.0–85.0
ADHD‐NF	13	Male	7	13.3	1.2	11.9–14.8
		Female	6	13.4	0.8	12.1–14.2
Indiana (2)	66	Male	26	40.2	15.3	19.0–65.0
		Female	40	39.4	14.1	20.0–65.0
Sydney MAS	523	Male	236	78.3	4.6	70.3–89.8
		Female	287	78.5	4.7	70.5–90.1
OADS (1)	118	Male	39	73.8	5.5	65.0–84.0
		Female	79	70.4	5.6	65.0–84.0
Cardiff	318	Male	89	28.1	7.8	19.0–57.0
		Female	229	24.2	7.0	18.0–58.0
CEG	32	Male	32	15.6	1.7	13.0–19.0
NYU	51	Male	31	30.2	7.7	18.8–46.0
		Female	20	31.4	10.3	19.8–51.9
CLiNG	321	Male	131	25.5	5.4	19.0–58.0
		Female	190	24.9	5.1	18.0–57.0
NTR (1)	112	Male	42	28.5	8.0	19.0–56.0
		Female	70	37.0	10.5	19.0–57.0
NTR (2)	30	Male	11	28.4	3.6	22.0–33.0
		Female	19	28.6	9.8	1.0–42.0
NTR (3)	37	Male	14	15.1	1.5	12.0–17.0
		Female	23	14.5	1.4	11.0–18.0
Indiana (2) + (3)	201	Male	97	21.6	14.4	6.0–79.0
		Female	104	33.0	22.8	7.0–87.0
BIG	1291	Male	553	25.1	9.3	18.0–71.0
		Female	738	23.3	6.9	18.0–66.0
OADS (2)	35	Male	15	70.1	5.7	65.0–81.0
		Female	20	67.4	3.8	65.0–78.0
OADS (3)	153	Male	59	70.3	4.2	65.0–81.0
		Female	94	69.7	4.6	65.0–81.0
OADS (4)	108	Male	30	69.8	4.5	65.0–85.0
		Female	78	70.1	4.9	65.0–89.0
MHRC	52	Male	52	22.3	2.9	16.1–27.6
BRAINSCALE	277	Male	146	10.1	1.5	9.0–15.0
		Female	131	9.9	1.2	9.0–14.1
Leiden	611	Male	299	16.2	4.7	8.3–28.1
		Female	312	16.9	4.9	8.4–28.9
IMAGEN	1964	Male	952	14.5	0.4	13.2–15.7
		Female	1012	14.5	0.4	13.3–16.0
ENIGMA‐HIV	175	Male	175	38.8	6.5	29.0–50.0
UMCU	172	Male	84	40.2	16.5	18.0–80.0
		Female	88	39.2	17.9	18.0–84.0

### 2.2 Imaging data acquisition and processing

For definition of all brain measures, whole‐brain T1‐weighted anatomical scan were included. Detailed information on scanner model and image acquisition parameters for each site can be found in Supplemental Table 1. T1 weighted scans were processed at the cohort level, where subcortical segmentation and cortical parcellation were performed by running the T1‐weighted images in FreeSurfer using versions 4.1, 5.1, 5.3 or 6.0 (see Supplemental Table 1 for specifications per site). This software suite is well validated and widely used, and documented and freely available online (surfer.nmr.mgh.harvard.edu). The technical details of the automated reconstruction scheme are described elsewhere (Dale, Fischl, and Sereno 1999; Fischl et al. 1999, 2002). The outcome variables included volumes of seven subcortical structures: accumbens, caudate, pallidum, putamen, amygdala, hippocampus, and thalamus (Fischl et al. 2002), and cortical surface area and thickness measures (Dale et al. 1999; Fischl et al. 1999) of 68 regions of the cerebral cortex (Desikan‐Killiany atlas) (Desikan et al. 2006). Quality control was also implemented at the cohort level following detailed protocols (http://enigma.ini.usc.edu/protocols/imaging‐protocols). The statistical analyses included 13,696 participants for subcortical volumes, 11,338 for surface area measures, and 12,533 participants for cortical thickness analysis.

### 2.3 Statistical analysis

Statistical analyses were performed using R Statistical Software. The complete scripts are available in the Appendix. In brief, we first adjusted all brain structure variables for cohort, field strength and FreeSurfer version effects. As age ranges differed for each cohort this was done in two steps: initially, a linear model was used to account for cohort effects and non‐linear age effects, using a third‐degree polynomial function. Next, random forest regression modelling (Breiman 2001) was used to additionally account for field strength and FreeSurfer version. See Supplemental Figure 1 for adjusted values. This was implemented in the R package *randomForest*, which can accommodate models with interactions and non‐linear effects.

### 2.4 Mean differences

Mean sex differences in brain structure variables were tested using t‐tests (FDR corrected, see (Benjamini and Hochberg 1995)) and effect sizes were estimated using Cohen's *d*‐value. A negative effect size indicates that the mean was higher in females, and a positive effect size indicates it was higher in males. The brain structure variables were adjusted for age and covariates described above. Graphs were created with R package ggseg (Mowinckel and Vidal‐Pineiro, 2019).

### 2.5 Variance ratio

Variance differences between males and females were examined, after accounting for age and other covariates as described above. Fisher's variance ratio (VR) was estimated by dividing variance measures for males and females. VR was log transformed to account for VR bias (Katzman and Alliger 1992; Lehre et al. 2009). Letting *y*_*i*_ denote the observed outcome for observation number *i* and *y*^_*i*_ its predicted outcome, the residuals were then formed:

ri=yi−y^i

The residual variance *Var*_males_ and *Var*_females_ were computed separately for males and females, and used to form the test statistic

$$T={\mathrm{Var}}_{\text{males}}/{\mathrm{Var}}_{\text{females}}$$

For each outcome, a permutation test of the hypothesis that the sex specific standard deviations were equal, was performed. This was done by random permutation of the sex variable among the residuals. Using *β* permutations, the *p*‐value for the *k*‐th outcome measure was computed as

$$p_{k}=\sum_{b=1}^{B}I\left(T_{b}>T\right)/B$$

where *I*(*T*_*b*_ ≥ *T*) is an indicator function that is 1 when *T*_*b*_ ≥ *T*, and 0 otherwise. Thus, the *p*‐value is the proportion of permuted test statistics (*T*_*b*_) that were greater than the observed value *T* of the test statistic above. Here *B* was set to 10,000. FDR corrected values are reported as significant.

### 2.6 Shift Function

To assess the nature of the variability difference between males and females, shift functions were estimated for each brain measure that showed significant variance differences between males and females using quantile regression forests (Meinshausen 2006; Rousselet, Pernet, and Wilcox 2017), implemented in the R package quantregForest (see Wierenga et al. 2018) for a similar approach). First, as described above, brain measures were accounted for site, age, field strength and FreeSurfer version. Next, quantile distribution functions were estimated for males and females separately after aligning the distribution means. Let *q* be a probability between 0 and 1. The quantile function specifies the values at which the volume of a brain measure will be at or below any given *q*. The quantile function for males is given as *Q*(*q*| *males*) and for females as *Q*(*q*|*females*). The quantile distance function is then defined as:

$$D\left(q\right)=Q\left(q|\text{males}\right)-Q\left(q|\text{females}\right)$$

A bootstrap method was used to estimate the standard error of the quantile difference functions, which was used to form approximate 95% confidence intervals. If the quantile distance function is a straight‐line parallel to the *x* axis, this indicates a stable difference between the sexes across the distribution and thus no detectable difference in variability. A positive slope indicates greater male variance. More specifically, this would indicate that the males with the largest values have relatively larger values than females with the largest values, and males with the smallest values are relatively smaller values than the females with the smallest values. A negative slope of the quantile distance function would indicate larger variability in females at both ends of the distribution.

### 2.7 Variance change with age

To study whether the sex differences in variance are stable across the age range we used the residuals of the predicted outcome measure and each individual *i*:

ri=∣yi−y^i∣

The absolute value of *r*_*i*_ was then used in a regression model. It was next explored whether there was a significant (FDR corrected) age by sex interaction effect using a linear model 1 and quadratic model 2:

$$y_{i}=\mathrm{Ag}e_{i}*\mathrm{se}x_{i}+\text{erro}r_{i}\;\left(\text{model}\;1\right)$$

$$y_{i}={\mathrm{Age}}_{i}^{2}*\mathrm{se}x_{i}+\text{erro}r_{i}\;\left(\text{model}\;2\right)$$

### 2.8 Anatomical correlation analysis

Inter‐regional anatomical associations were assessed by defining the correlation between two brain structures, after accounting for age and other covariates as described above. Anatomical correlation matrices were estimated as previously applied in several structural MRI studies for males and females separately (see e.g. Baaré et al. 2001; Lerch et al. 2006). Next, the anatomical correlation matrix for females was subtracted from the anatomical correlation matrix for males, yielding a difference matrix.

Thus, the Pearson correlation coefficient between any two regions *i* and *j* was assessed for males and females separately. This produced two group correlation matrices *M*_*ij*_ and *F*_*ij*_ where *i*, *j*, = 1, 2, . …, *N*, where *N* is the number of brain regions.

Sex specific means and standard deviations were removed by performing sex specific standardization. The significance of the differences between ***M***_***ij***_ and ***F***_***ij***_ was assessed by the difference in their Fisher's ***z***‐transformed values, and *p*‐values were computed using permutations. Whether these significantly differed between the sexes was tested using a Chi‐square test.

## 3 RESULTS

### 3.1 Sex differences in mean and variance

**FIGURE 1:**
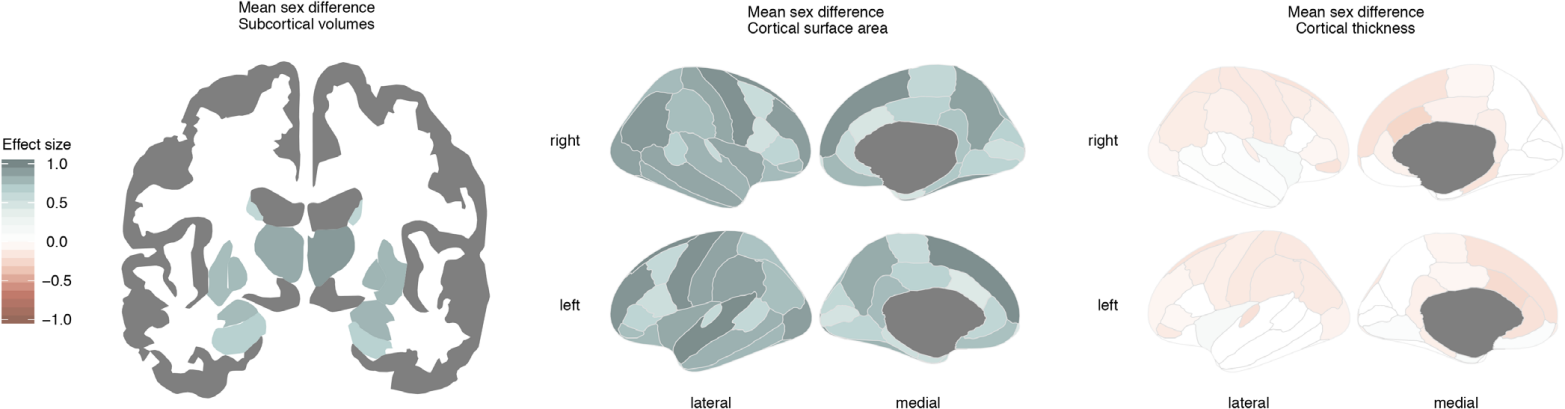
Sex differences in volumetric measures of subcortical volumes (left), cortical surface area (center), and cortical thickness (right). Shown are effect sizes (Cohen's d‐value) of FDR corrected mean sex differences. Greater mean values for males are displayed in blue, greater mean values for females are displayed in red. Darker colors indicate larger effect sizes

All brain measures were adjusted for cohort, field strength, FreeSurfer version and (non‐linear) age. As a background analysis, we first assessed whether brain structural measures showed mean differences between males and females to align our findings to previous reports (Figure 1, Table 2). All subcortical volumes were significantly larger in males, with effect sizes (Cohen's *d*‐values) ranging from 0.41 (left accumbens) to 0.92 (right thalamus), and an average effect size of 0.7. In follow‐up analyses with total brain volume as an additional covariate we found a similar pattern, although effect sizes were smaller (Supplemental Table S2A). Also for cortical surface area, all regions showed significantly larger values in males than females, with effect sizes ranging from 0.42 (left caudal anterior cingulate area) to 0.97 (left superior temporal area), on average 0.71. When total surface area was included as an additional covariate, a similar pattern was observed, although effect sizes were smaller (Supplemental Table S2B). Cortical thickness showed significant mean sex differences in 43 (out of 68) regions, of which 38 regions showed larger thickness values in females than males. These were mostly frontal and parietal regions. The largest effect size, however, was only 0.12 (right caudal anterior cingulate cortex). When total average cortical thickness was included as an additional covariate, nine regions showed a male advantage that was not observed in the raw data analysis, and six of the 38 regions showing female advantage did not reach significance (Supplemental Table S2C).

**TABLE 2:** Sex differences in mean and variance

(a) Subcortical volume	Female (*n* = 7141)	Male (*n* = 6555)	Mean difference test		Variance	Ratio test
	M	M	*p*	Cohen's *d*	VR	*p*
Left thal	‐328.287	357.024	**	0.840	0.237	**
Right thal	‐317.358	345.963	**	0.918	0.357	**
Left caud	‐139.573	152.488	**	0.609	0.150	**
Right caud	‐147.366	160.706	**	0.625	0.147	**
Left put	‐237.405	257.178	**	0.757	0.197	**
Right put	‐233.415	252.623	**	0.786	0.220	**
Left pal	‐86.166	93.761	**	0.768	0.317	**
Right pal	‐74.910	81.507	**	0.793	0.339	**
Left hippo	‐137.976	149.409	**	0.673	0.173	**
Right hippo	‐134.745	145.724	**	0.669	0.232	**
Left amyg	‐73.754	80.305	**	0.765	0.154	**
Right amyg	‐80.242	87.372	**	0.790	0.216	**
Left accumb	‐22.255	24.369	**	0.414	0.168	**
Right accumb	‐22.755	24.685	**	0.454	0.119	**

(b) Surface area	Female (*n* = 6243)	Male (*n* = 5092)	Mean difference test		Variance	Ratio test
	M	M	*p*	Cohen's *d*	VR	*p*
Left bankssts	‐45.976	56.715	**	0.596	0.282	**
Left caudalanteriorcingulate	‐25.875	31.956	**	0.420	0.131	**
Left caudalmiddlefrontal	‐100.326	123.509	**	0.589	0.163	**
Left cuneus	‐55.069	67.958	**	0.605	0.188	**
Left entorhinal	‐19.379	23.824	**	0.540	0.310	**
Left fusiform	‐142.081	174.977	**	0.794	0.240	**
Left inferiorparietal	‐203.760	250.694	**	0.751	0.288	**
Left inferiortemporal	‐158.709	195.821	**	0.778	0.193	**
Left isthmuscingulate	‐54.544	67.228	**	0.765	0.326	**
Left lateraloccipital	‐229.910	284.223	**	0.893	0.240	**
Left lateralorbitofrontal	‐93.815	115.782	**	0.771	0.194	**
Left lingual	‐114.132	141.130	**	0.630	0.197	**
Left medialorbitofrontal	‐76.336	94.318	**	0.741	0.288	**
Left middletemporal	‐139.909	172.666	**	0.808	0.227	**
Left parahippocampal	‐24.273	30.139	**	0.522	0.330	**
Left paracentral	‐46.588	57.790	**	0.578	0.303	**
Left parsopercularis	‐63.862	78.461	**	0.536	0.350	**
Left parsorbitalis	‐27.703	34.060	**	0.755	0.223	**
Left parstriangularis	‐55.836	68.926	**	0.633	0.262	**
Left pericalcarine	‐48.359	58.895	**	0.485	0.151	**
Left postcentral	‐176.934	217.762	**	0.867	0.286	**
Left posteriorcingulate	‐50.597	62.161	**	0.651	0.253	**
Left precentral	‐207.652	255.826	**	0.949	0.319	**
Left precuneus	‐163.276	200.728	**	0.834	0.266	**
Left rostralanteriorcingulate	‐40.967	50.637	**	0.619	0.160	**
Left rostralmiddlefrontal	‐297.267	365.653	**	0.934	0.261	**
Left superiorfrontal	‐330.564	406.757	**	0.962	0.269	**
Left superiorparietal	‐202.642	249.403	**	0.730	0.241	**
Left superiortemporal	‐177.562	218.916	**	0.970	0.262	**
Left supramarginal	‐205.547	254.230	**	0.877	0.304	**
Left frontalpole	‐6.671	8.241	**	0.439	0.249	**
Left temporalpole	‐15.185	18.664	**	0.557	0.224	**
Left transversetemporal	‐19.898	24.463	**	0.585	0.239	**
Left insula	‐84.765	104.782	**	0.847	0.250	**
Right bankssts	‐42.654	52.655	**	0.662	0.261	**
Right caudalanteriorcingulate	‐31.929	39.489	**	0.465	0.275	**
Right caudalmiddlefrontal	‐95.924	117.705	**	0.563	0.225	**
Right cuneus	‐61.606	75.541	**	0.668	0.213	**
Right entorhinal	‐16.941	20.615	**	0.467	0.339	**
Right fusiform	‐155.696	191.647	**	0.900	0.225	**
Right inferiorparietal	‐278.411	342.870	**	0.920	0.325	**
Right inferiortemporal	‐157.460	193.922	**	0.827	0.187	**
Right isthmuscingulate	‐47.046	57.740	**	0.723	0.314	**
Right lateraloccipital	‐227.765	282.023	**	0.876	0.279	**
Right lateralorbitofrontal	‐99.594	122.823	**	0.765	0.234	**
Right lingual	‐110.640	136.478	**	0.644	0.225	**
Right medialorbitofrontal	‐70.180	86.695	**	0.777	0.203	**
Right middletemporal	‐155.924	192.222	**	0.857	0.224	**
Right parahippocampal	‐30.721	37.810	**	0.708	0.357	**
Right paracentral	‐57.941	71.375	**	0.609	0.349	**
Right parsopercularis	‐53.895	65.892	**	0.506	0.312	**
Right parsorbitalis	‐35.086	43.159	**	0.771	0.197	**
Right parstriangularis	‐69.557	85.138	**	0.634	0.252	**
Right pericalcarine	‐56.327	68.894	**	0.528	0.145	**
Right postcentral	‐168.595	208.307	**	0.851	0.278	**
Right posteriorcingulate	‐52.836	65.327	**	0.662	0.237	**
Right precentral	‐216.995	267.894	**	0.950	0.341	**
Right precuneus	‐184.909	228.043	**	0.878	0.248	**
Right rostralanteriorcingulate	‐33.179	41.005	**	0.576	0.221	**
Right rostralmiddlefrontal	‐294.685	363.055	**	0.898	0.228	**
Right superiorfrontal	‐325.198	400.002	**	0.939	0.258	**
Right superiorparietal	‐205.624	252.962	**	0.765	0.216	**
Right superiortemporal	‐132.506	163.787	**	0.800	0.243	**
Right supramarginal	‐168.426	207.920	**	0.754	0.285	**
Right frontalpole	‐9.712	11.996	**	0.481	0.194	**
Right temporalpole	‐11.097	13.725	**	0.422	0.228	**
Right transversetemporal	‐14.315	17.686	**	0.564	0.194	**
Right insula	‐95.695	117.482	**	0.863	0.238	**

(c) Thickness	Female (*n* = 6620)	Male (*n* = 5913)	Mean difference test		Variance	Ratio test
	M	M	*p*	Cohen's *d*	VR	*p*
Left bankssts	0.001	‐0.001	n.s.	0.011	0.039	**
Left caudalanteriorcingulate	0.026	‐0.028	**	0.213	‐0.042	n.s.
Left caudalmiddlefrontal	0.008	‐0.008	**	0.103	0.061	*
Left cuneus	0.000	0.000	n.s.	0.001	0.050	*
Left entorhinal	‐0.013	0.015	**	0.084	0.023	n.s.
Left fusiform	0.001	‐0.001	n.s.	0.016	0.022	n.s.
Left inferiorparietal	0.009	‐0.009	**	0.128	0.092	**
Left inferiortemporal	‐0.002	0.003	n.s.	0.027	0.004	n.s.
Left isthmuscingulate	0.009	‐0.009	**	0.088	‐0.007	**
Left lateraloccipital	0.005	‐0.005	**	0.074	0.079	**
Left lateralorbitofrontal	‐0.002	0.003	n.s.	0.036	0.101	**
Left lingual	‐0.003	0.004	**	0.058	0.040	n.s.
Left medialorbitofrontal	‐0.004	0.006	**	0.058	0.027	n.s.
Left middletemporal	‐0.003	0.004	n.s.	0.037	0.093	*
Left parahippocampal	0.015	‐0.016	**	0.098	0.016	n.s.
Left paracentral	0.006	‐0.005	**	0.067	0.030	**
Left parsopercularis	‐0.002	0.003	n.s.	0.027	0.087	**
Left parsorbitalis	0.013	‐0.014	**	0.120	0.071	**
Left parstriangularis	0.004	‐0.004	*	0.049	0.084	**
Left pericalcarine	0.000	0.001	n.s.	0.006	0.043	**
Left postcentral	0.008	‐0.009	**	0.133	0.078	**
Left posteriorcingulate	0.004	‐0.004	**	0.052	0.080	**
Left precentral	0.007	‐0.007	**	0.097	0.112	**
Left precuneus	0.000	0.000	n.s.	0.002	0.041	**
Left rostralanteriorcingulate	0.020	‐0.021	**	0.170	‐0.046	n.s.
Left rostralmiddlefrontal	0.005	‐0.004	**	0.061	0.112	**
Left superiorfrontal	0.013	‐0.014	**	0.168	0.048	n.s.
Left superiorparietal	0.009	‐0.009	**	0.136	0.098	**
Left superiortemporal	‐0.001	0.001	n.s.	0.014	0.052	**
Left supramarginal	0.009	‐0.009	**	0.126	0.064	**
Left frontalpole	0.015	‐0.016	**	0.100	0.036	n.s.
Left temporalpole	0.004	‐0.004	n.s.	0.023	0.027	n.s.
Left transversetemporal	0.020	‐0.021	**	0.177	0.018	n.s.
Left insula	‐0.009	0.011	**	0.121	0.049	n.s.
Right bankssts	‐0.001	0.002	n.s.	0.016	0.064	**
Right caudalanteriorcingulate	0.027	‐0.030	**	0.242	‐0.029	n.s.
Right caudalmiddlefrontal	0.008	‐0.009	**	0.109	0.019	**
Right cuneus	0.003	‐0.002	n.s.	0.034	0.027	*
Right entorhinal	0.005	‐0.005	n.s.	0.028	0.026	n.s.
Right fusiform	0.001	0.000	n.s.	0.008	0.029	n.s.
Right inferiorparietal	0.008	‐0.008	**	0.110	0.103	**
Right inferiortemporal	0.000	0.001	n.s.	0.003	0.032	n.s.
Right isthmuscingulate	0.010	‐0.010	**	0.099	‐0.038	**
Right lateraloccipital	0.004	‐0.004	**	0.057	0.078	**
Right lateralorbitofrontal	0.003	‐0.003	n.s.	0.036	0.074	**
Right lingual	‐0.002	0.003	n.s.	0.036	0.036	n.s.
Right medialorbitofrontal	0.003	‐0.003	n.s.	0.033	0.056	n.s.
Right middletemporal	‐0.003	0.004	*	0.047	0.065	**
Right parahippocampal	0.021	‐0.023	**	0.162	0.028	n.s.
Right paracentral	0.004	‐0.004	**	0.055	0.065	**
Right parsopercularis	0.000	0.000	n.s.	0.001	0.037	**
Right parsorbitalis	0.018	‐0.019	**	0.164	0.026	n.s.
Right parstriangularis	0.004	‐0.004	**	0.053	0.008	**
Right pericalcarine	0.001	‐0.001	n.s.	0.017	0.020	n.s.
Right postcentral	0.009	‐0.009	**	0.135	0.009	**
Right posteriorcingulate	0.007	‐0.007	**	0.082	0.013	**
Right precentral	0.008	‐0.009	**	0.119	0.084	**
Right precuneus	‐0.001	0.002	n.s.	0.018	0.063	**
Right rostralanteriorcingulate	0.009	‐0.010	**	0.080	0.055	n.s.
Right rostralmiddlefrontal	0.006	‐0.006	**	0.078	0.085	**
Right superiorfrontal	0.013	‐0.013	**	0.165	0.065	*
Right superiorparietal	0.008	‐0.009	**	0.132	0.065	**
Right superiortemporal	‐0.003	0.004	*	0.042	0.073	**
Right supramarginal	0.006	‐0.007	**	0.086	0.096	**
Right frontalpole	0.021	‐0.022	**	0.140	0.012	n.s.
Right temporalpole	‐0.006	0.007	*	0.038	0.023	n.s.
Right transversetemporal	0.011	‐0.031	**	0.095	0.101	*
Right insula	‐0.008	0.010	**	0.107	0.092	**

* *p* < 0.05, ** *p* < 0.01, both after FDR correction.

**FIGURE 2:**
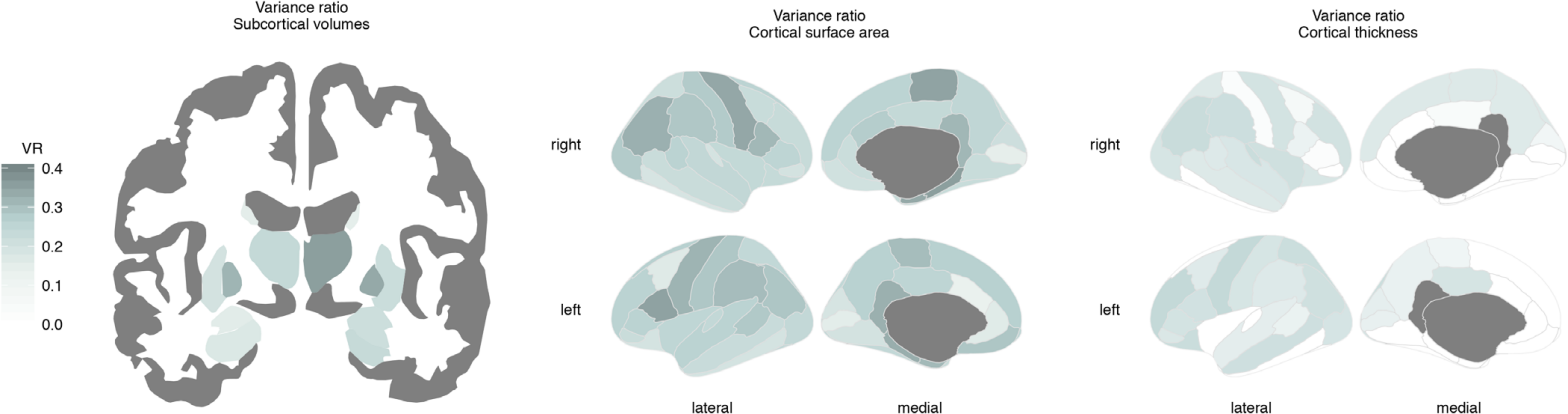
Sex differences in variance ratio for subcortical volumes (Left), cortical surface area (center), and cortical thickness (right). Shown are log transformed variance ratios, where significant larger variance ratio for males than females is displayed in blue ranging from 0 to 1. Darker colors indicate a larger variance ratio

We then tested for sex differences in variance of brain structure, adjusted for cohort, field strength, FreeSurfer version and (non‐linear) age (Figure 2, Tables 2). All subcortical volumes had significantly greater variance in males than females. Log transformed variance ratios ranged from 0.12 (right accumbens) to 0.36 (right pallidum), indicating greater variance in males than females. Similar results were also observed when total brain volume was taken into account (Supplemental Table S2A). Cortical surface area also showed significantly greater variance in males for all regions: variance ratios ranged from 0.13 (left caudal anterior cingulate cortex) to 0.36 (right parahippocampal cortex). This pattern was also observed when total surface area was included in the model (Supplemental Table S2B). Cortical thickness showed significantly greater male variance in 41 out of 68 regions, with the greatest variance ratio being 0.11 (left precentral cortex). Notably, 37 of these 41 regions did not show significantly larger mean thickness values in males. When additionally accounting for total average thickness, we found greater male variance in 39 regions and greater females variance in 5 regions. Also here, significant variance ratios were present in the absence of mean sex differences (Supplemental Table S2C).

Next, we directly tested whether the regions showing larger variance effects were also those showing larger mean differences, by correlating the variance ratios with the vector of *d*‐values (Supplemental Figure 2). There was a significant association for subcortical volumes (r (12) = 0.7, *p*‐value = .005), but no significant relation for regional cortical surface area (r (66) = 0.18, *p*‐value = .14), or thickness (r (66) = ‐0.21, *p*‐value = .09).

### 3.2 Greater variance in males at upper and lower tails

**FIGURE 3:**
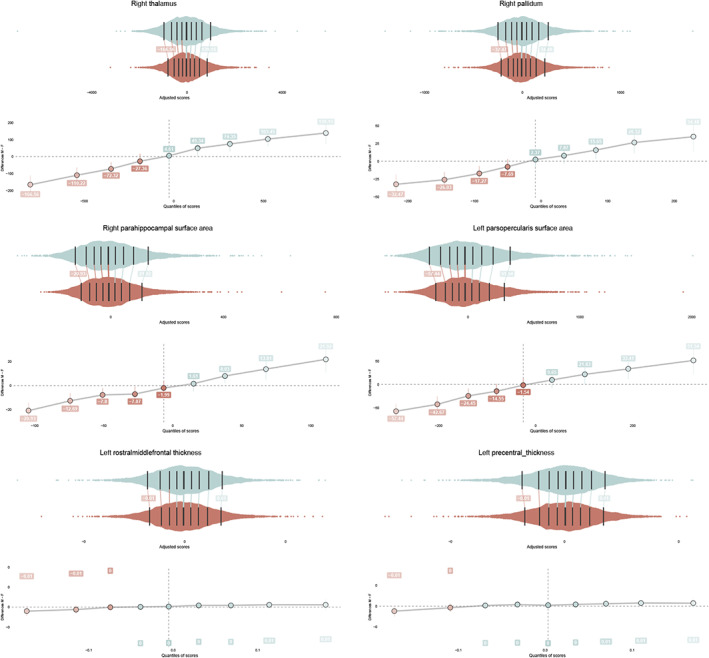
Jittered marginal distribution scatterplots are displayed together with their shift function for the top three variance ratio effects of subcortical volumes (top), cortical surface area (middle) and cortical thickness (right). The central, darkest line on each distribution is the median, note that main sex effects are removed. The other lines mark the deciles of each distribution. The shift values are included, which refer to the number of units that the male (upper) distribution would have to be shifted to match the female (lower) distribution. Confidence intervals are included for each of these shift values

In order to characterise how the distributions of males and females differ, quantiles were compared using a shift function (Rousselet et al. 2017). As in the previous models, brain measures were adjusted for cohort, field strength, FreeSurfer version and age. In addition, the distribution means were aligned. Results showed greater male variance at both upper and lower tails for regions that showed significant variance differences between males and females. The top three variance ratio effects for subcortical volume, cortical surface area and cortical thickness are shown in Figure 3.

### 3.3 Variance differences between sexes across age

**FIGURE 4:**
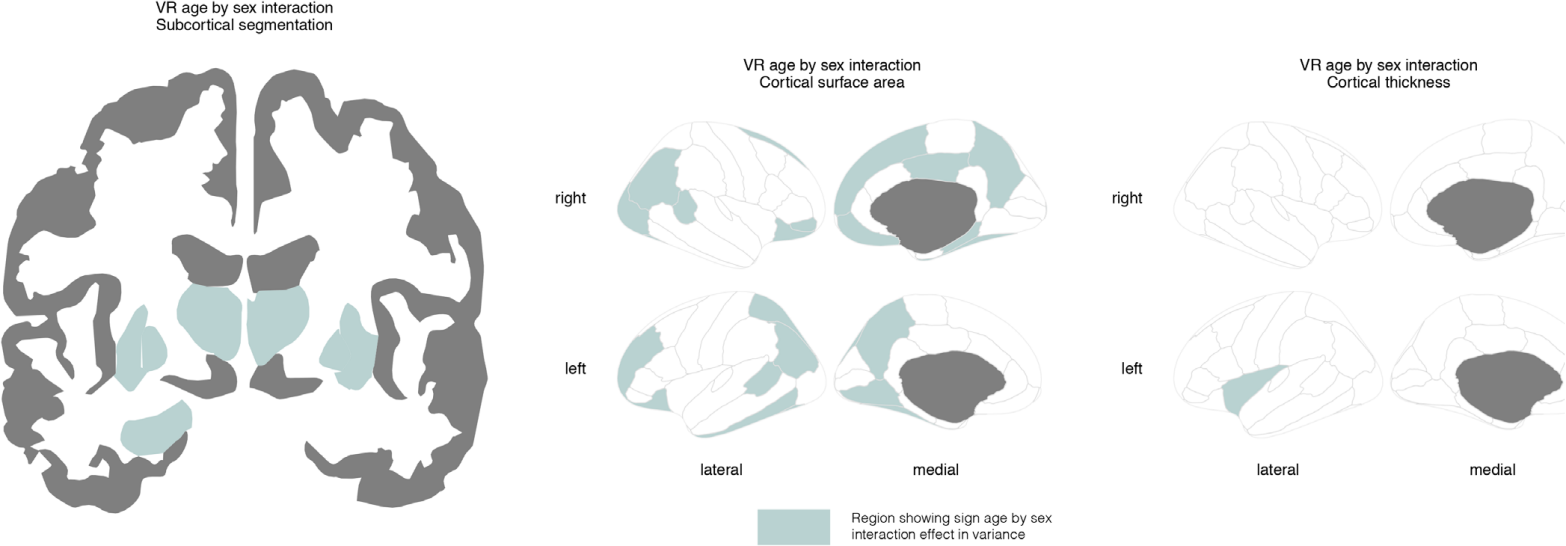
Regions where sex differences in variability of brain structure interacted with age displayed for subcortical volumes (left), cortical surface area (center), and cortical thickness (right)

**FIGURE 5:**
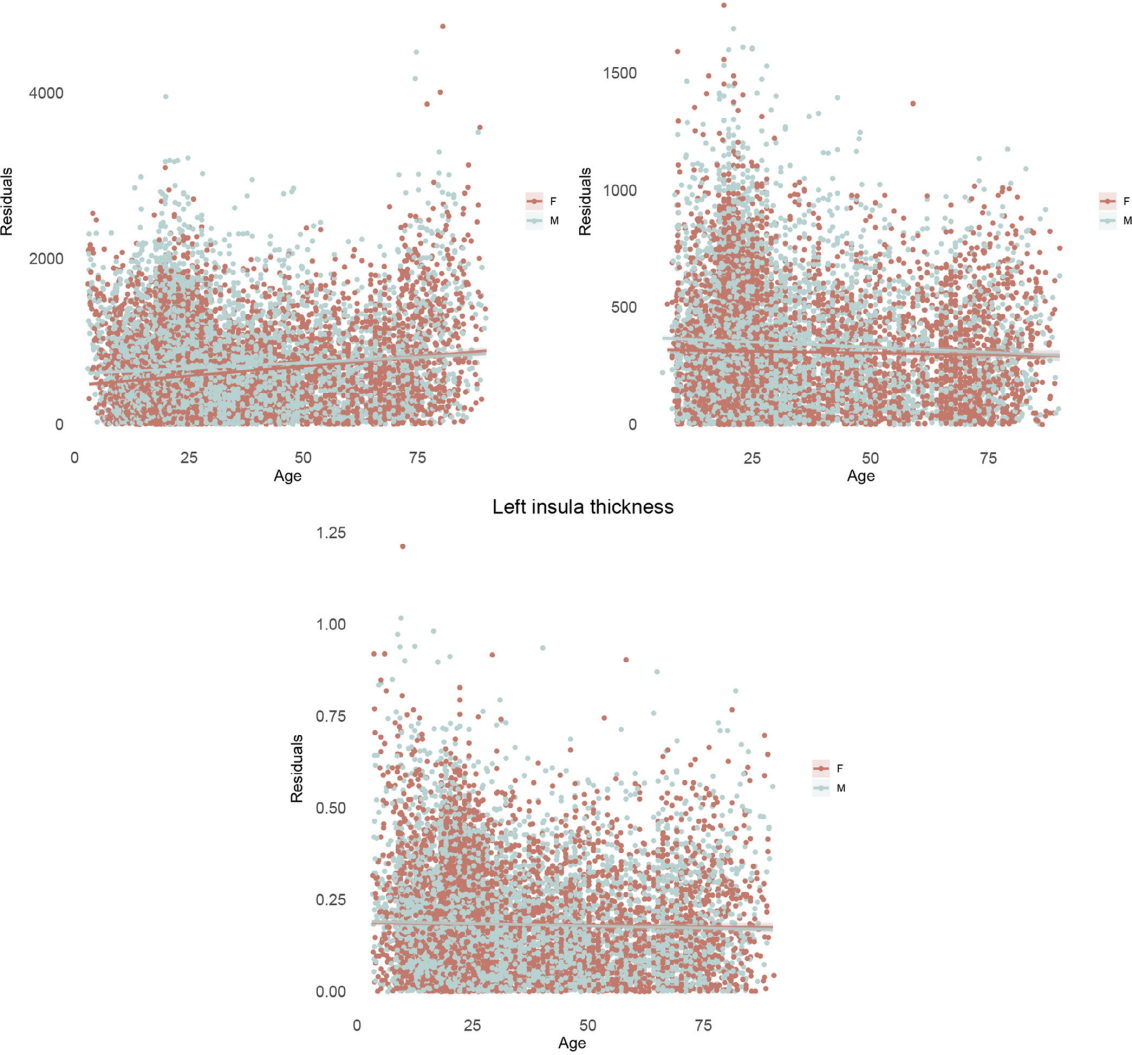
Sex differences in variability interacted with age in 50% of the subcortical volumes, 30% of the surface area measures, and only one thickness measure. Three representative results are shown: right thalamus volume (top left), surface area of the right parahippocampal gyrus (top right) and thickness of the left insula (bottom center). Absolute residual values are modeled across the age range. Effects showed larger male than female variance in the younger age group, this effect attenuated with increasing age

We next tested whether the sex differences in variance interacted with age (Figure 4 and supplemental Figure 3). In this set of analyses, brain measures were adjusted for cohort, field strength, and FreeSurfer version. For 50% of the subcortical volume measures there was a significant interaction, specifically for the bilateral thalami, bilateral putamen, bilateral pallidum and the left hippocampus (Table 3, Figure 5). Cortical surface area showed significant interaction effects in 30% of the cortical regions (Table 3, Figure 5). In both cases, younger individuals tended to show greater sex differences in variance than older individuals. For cortical thickness, an interaction with age was detected only in the left insula (Table 3, Figure 5). This region showed greater male than female variance in the younger age group, whereas greater female variance was observed in older individuals.

**TABLE 3:** Variance differences between sexes across age

(a) Subcortical	Intercept	*SE*	*p*	Age	*SE*	*p*	Sex	*SE*	P	Sex by age	*SE*	*p*
Left thal	587.987	6.178	**	9398.523	652.185	**	60.310	9.199	**	‐3107.885	979.201	**
Right thal	515.416	5.524	**	6424.232	583.119	**	82.380	8.225	**	‐3102.267	875.503	**
Left caud	361.790	3.729	**	879.545	393.693	*	28.152	5.553	**	270.769	591.096	n.s.
Right caud	371.773	3.785	**	1290.352	399.567	**	31.395	5.636	**	‐561.719	599.915	n.s.
Left put	495.399	5.150	**	4435.730	543.701	**	54.586	7.669	**	‐2966.533	816.321	**
Right put	460.842	4.887	**	5622.177	515.939	**	51.687	7.277	**	‐3853.454	774.638	**
Left pal	165.039	1.816	**	837.030	191.768	**	26.852	2.705	**	‐784.363	287.923	*
Right pal	140.799	1.598	**	910.463	168.695	**	26.247	2.379	**	‐850.994	253.281	**
Left hippo	309.722	3.308	**	2755.892	349.231	**	31.626	4.926	**	‐1375.500	524.341	*
Right hippo	305.607	3.264	**	2615.969	344.571	**	35.732	4.860	**	‐890.970	517.345	n.s.
Left amyg	148.932	1.598	**	1378.267	168.734	**	13.800	2.380	**	‐233.236	253.340	n.s.
Right amyg	154.218	1.645	**	1621.298	173.675	**	16.477	2.450	**	‐540.141	260.758	n.s.
Left accumb	82.473	0.875	**	442.922	92.410	**	7.382	1.303	**	‐136.472	138.746	n.s.
Right accumb	78.541	0.823	**	539.975	86.850	**	7.412	1.225	**	‐106.522	130.398	n.s.

Surface area	Intercept	*SE*	*p*	Age	*SE*	*p*	Sex	*SE*	*p*	Sex by age	*SE*	*p*
Left bankssts	127.133	1.376	**	‐437.616	142.554	**	16.563	2.056	**	‐574.105	219.785	*
Left caudalanteriorcingulate	104.209	1.113	**	‐302.669	115.254	**	4.299	1.663	**	‐277.614	177.695	n.s.
Left caudalmiddlefrontal	293.750	2.943	**	‐1359.284	304.791	**	21.272	4.397	**	‐660.300	469.918	n.s.
Left cuneus	154.129	1.607	**	‐360.698	166.430	*	13.158	2.401	**	‐330.457	256.596	n.s.
Left entorhinal	57.126	0.651	**	‐458.398	67.397	**	9.241	0.972	**	1.893	103.911	n.s.
Left fusiform	305.090	3.105	**	250.591	321.575	n.s.	35.738	4.639	**	‐2446.584	495.794	**
Left inferiorparietal	454.916	4.708	**	‐614.521	487.682	n.s.	63.459	7.035	**	‐2243.805	751.894	*
Left inferiortemporal	352.394	3.540	**	‐353.703	366.628	n.s.	31.482	5.289	**	‐1652.239	565.256	*
Left isthmuscingulate	116.771	1.249	**	‐32.188	129.411	n.s.	19.544	1.867	**	‐204.545	199.522	n.s.
Left lateraloccipital	438.089	4.474	**	‐1416.631	463.377	**	50.571	6.685	**	‐813.654	714.421	n.s.
Left lateralorbitofrontal	208.173	2.120	**	204.108	219.597	n.s.	20.633	3.168	**	‐1428.745	338.567	**
Left lingual	310.573	3.141	**	‐234.334	325.364	n.s.	29.898	4.694	**	‐1268.288	501.636	*
Left medialorbitofrontal	172.506	1.795	**	3.188	185.938	n.s.	23.450	2.682	**	‐213.946	286.673	n.s.
Left middletemporal	296.794	2.997	**	‐421.492	310.480	n.s.	31.627	4.479	**	‐1014.822	478.689	n.s.
Left parahippocampal	72.669	0.887	**	‐211.577	91.839	*	10.825	1.325	**	‐241.097	141.595	n.s.
Left paracentral	133.446	1.419	**	‐195.857	147.019	n.s.	19.139	2.121	**	‐171.708	226.670	n.s.
Left parsopercularis	193.582	2.113	**	‐540.023	218.880	*	31.583	3.158	**	‐459.911	337.462	n.s.
Left parsorbitalis	61.886	0.643	**	‐172.940	66.566	**	7.120	0.960	**	‐131.612	102.629	n.s.
Left parstriangularis	148.566	1.524	**	‐644.966	157.820	**	19.173	2.277	**	‐546.829	243.322	n.s.
Left pericalcarine	171.607	1.690	**	‐245.127	175.004	n.s.	13.803	2.525	**	‐283.583	269.815	n.s.
Left postcentral	340.927	3.572	**	‐1033.492	370.007	**	46.097	5.338	**	‐1240.366	570.466	n.s.
Left posteriorcingulate	130.459	1.363	**	‐176.189	141.217	n.s.	13.905	2.037	**	‐400.954	217.724	n.s.
Left precentral	360.893	3.926	**	‐1088.967	406.693	**	47.580	5.867	**	‐876.707	627.028	n.s.
Left precuneus	329.439	3.386	**	‐444.670	350.720	n.s.	44.718	5.060	**	‐1691.713	540.730	*
Left rostralanteriorcingulate	113.700	1.156	**	‐6.807	119.754	n.s.	7.691	1.728	**	‐80.447	184.632	n.s.
Left rostralmiddlefrontal	541.319	5.553	**	‐1574.677	575.208	**	63.888	8.298	**	‐2391.074	886.838	*
Left superiorfrontal	577.465	6.015	**	‐1306.494	623.063	*	75.007	8.988	**	‐2320.740	960.620	n.s.
Left superiorparietal	471.735	4.793	**	‐1198.240	496.487	*	57.076	7.162	**	‐2051.708	765.468	*
Left superiortemporal	308.552	3.215	**	‐864.236	333.037	**	40.486	4.804	**	‐1222.034	513.467	n.s.
Left supramarginal	392.296	4.082	**	‐1937.799	422.787	**	58.041	6.099	**	‐775.470	651.841	n.s.
Left frontalpole	25.431	0.265	**	‐114.432	27.425	**	3.212	0.396	**	‐7.992	42.283	n.s.
Left temporalpole	45.410	0.478	**	‐173.235	49.555	**	5.115	0.715	**	‐59.323	76.403	n.s.
Left transversetemporal	56.992	0.594	**	‐201.824	61.535	**	6.690	0.888	**	‐81.655	94.872	n.s.
Left insula	164.339	1.842	**	‐460.767	190.830	*	17.215	2.753	**	6.824	294.215	n.s.
Right bankssts	107.290	1.139	**	‐392.600	117.986	**	13.575	1.702	**	‐493.453	181.908	*
Right caudalanteriorcingulate	114.549	1.199	**	‐266.524	124.192	*	14.948	1.792	**	‐8.218	191.475	n.s.
Right caudalmiddlefrontal	288.671	2.929	**	‐1415.348	303.395	**	30.576	4.377	**	‐360.883	467.765	n.s.
Right cuneus	152.647	1.656	**	‐146.322	171.565	n.s.	16.151	2.475	**	‐436.462	264.513	n.s.
Right entorhinal	57.865	0.641	**	‐455.979	66.351	**	10.302	0.957	**	‐50.231	102.298	n.s.
Right fusiform	295.259	3.000	**	43.695	310.723	n.s.	32.408	4.483	**	‐1812.528	479.064	**
Right inferiorparietal	504.767	5.239	**	‐577.142	542.646	n.s.	82.015	7.828	**	‐2767.949	836.635	**
Right inferiortemporal	327.236	3.331	**	‐482.481	345.043	n.s.	28.512	4.978	**	‐1116.568	531.977	n.s.
Right isthmuscingulate	105.700	1.157	**	‐228.263	119.818	n.s.	16.311	1.729	**	‐192.830	184.732	n.s.
Right lateraloccipital	436.925	4.537	**	‐1283.916	469.975	**	58.726	6.780	**	‐1927.057	724.593	*
Right lateralorbitofrontal	220.527	2.284	**	236.472	236.616	n.s.	24.442	3.413	**	‐1470.759	364.808	**
Right lingual	289.568	3.001	**	‐299.806	310.855	n.s.	34.596	4.484	**	‐1128.138	479.266	n.s.
Right medialorbitofrontal	154.743	1.568	**	74.312	162.424	n.s.	15.452	2.343	**	‐964.430	250.420	**
Right middletemporal	309.733	3.171	**	‐517.078	328.408	n.s.	34.194	4.738	**	‐1188.068	506.329	n.s.
Right parahippocampal	70.171	0.781	**	‐155.100	80.940	n.s.	11.822	1.168	**	‐420.498	124.790	**
Right paracentral	156.024	1.669	**	‐273.907	172.868	n.s.	25.570	2.494	**	‐271.297	266.523	n.s.
Right parsopercularis	174.570	1.866	**	‐1036.595	193.296	**	25.454	2.789	**	‐231.029	298.018	n.s.
Right parsorbitalis	77.607	0.794	**	‐103.424	82.287	n.s.	7.160	1.187	**	‐311.879	126.867	*
Right parstriangularis	184.989	1.887	**	‐925.697	195.494	**	21.344	2.820	**	‐662.628	301.407	n.s.
Right pericalcarine	184.490	1.818	**	‐314.748	188.350	n.s.	13.276	2.717	**	‐264.356	290.392	n.s.
Right postcentral	330.886	3.494	**	‐1175.639	361.875	**	44.061	5.220	**	‐907.204	557.928	n.s.
Right posteriorcingulate	133.953	1.413	**	42.583	146.371	n.s.	14.739	2.112	**	‐695.150	225.670	*
Right precentral	374.619	4.131	**	‐1039.063	427.849	*	53.576	6.172	**	‐579.997	659.645	n.s.
Right precuneus	355.783	3.685	**	‐894.373	381.705	*	42.292	5.507	**	‐1788.652	588.501	*
Right rostralanteriorcingulate	97.009	1.005	**	198.486	104.078	n.s.	10.668	1.501	**	‐140.756	160.464	n.s.
Right rostralmiddlefrontal	560.924	5.691	**	‐2015.333	589.514	**	60.682	8.504	**	‐1467.830	908.895	n.s.
Right superiorfrontal	586.059	6.054	**	‐748.583	627.121	n.s.	72.274	9.047	**	‐3613.685	966.876	**
Right superiorparietal	453.081	4.716	**	‐1983.725	488.528	**	49.530	7.048	**	42.170	753.197	n.s.
Right superiortemporal	281.023	2.898	**	‐481.481	300.133	n.s.	31.844	4.330	**	‐1005.995	462.736	n.s.
Right supramarginal	376.538	3.839	**	‐1315.029	397.627	**	51.001	5.736	**	‐1362.209	613.049	n.s.
Right frontalpole	34.322	0.352	**	‐93.541	36.451	*	2.974	0.526	**	‐112.046	56.199	n.s.
Right temporalpole	44.173	0.457	**	‐144.791	47.330	**	5.067	0.683	**	‐32.370	72.972	n.s.
Right transversetemporal	43.342	0.436	**	‐122.601	45.112	**	4.348	0.651	**	‐76.872	69.553	n.s.
Right insula	185.386	1.947	**	167.564	201.684	n.s.	22.970	2.910	**	‐270.419	310.950	n.s.

Thickness	Intercept	*SE*	*p*	Age	*SE*	*p*	Sex	*SE*	*p*	Sex by age	*SE*	*p*
Left bankssts	0.138	0.001	**	0.012	0.150	n.s.	0.002	0.002	n.s.	0.345	0.217	n.s.
Left caudalanteriorcingulate	0.204	0.002	**	1.405	0.217	**	‐0.005	0.003	n.s.	0.207	0.314	n.s.
Left caudalmiddlefrontal	0.119	0.001	**	0.375	0.131	**	0.002	0.002	n.s.	‐0.108	0.190	n.s.
Left cuneus	0.108	0.001	**	‐0.194	0.118	n.s.	0.003	0.002	n.s.	‐0.386	0.171	n.s.
Left entorhinal	0.263	0.003	**	0.348	0.288	n.s.	0.001	0.004	n.s.	‐0.414	0.417	n.s.
Left fusiform	0.114	0.001	**	0.484	0.125	**	0.000	0.002	n.s.	‐0.340	0.181	n.s.
Left inferiorparietal	0.109	0.001	**	0.329	0.122	**	0.005	0.002	**	0.023	0.176	n.s.
Left inferiortemporal	0.128	0.001	**	0.515	0.138	**	0.000	0.002	n.s.	‐0.327	0.199	n.s.
Left isthmuscingulate	0.165	0.002	**	0.491	0.175	**	‐0.003	0.002	n.s.	‐0.076	0.254	n.s.
Left lateraloccipital	0.096	0.001	**	0.132	0.106	n.s.	0.004	0.001	**	0.057	0.154	n.s.
Left lateralorbitofrontal	0.124	0.001	**	0.212	0.138	n.s.	0.006	0.002	**	‐0.438	0.201	n.s.
Left lingual	0.099	0.001	**	0.343	0.109	**	0.001	0.001	n.s.	‐0.308	0.157	n.s.
Left medialorbitofrontal	0.135	0.001	**	0.067	0.150	n.s.	0.004	0.002	n.s.	‐0.425	0.217	n.s.
Left middletemporal	0.129	0.001	**	0.493	0.140	**	0.004	0.002	*	‐0.012	0.203	n.s.
Left parahippocampal	0.248	0.002	**	0.441	0.254	n.s.	0.002	0.003	n.s.	‐0.372	0.368	n.s.
Left paracentral	0.126	0.001	**	0.321	0.138	*	0.003	0.002	n.s.	‐0.017	0.199	n.s.
Left parsopercularis	0.123	0.001	**	0.497	0.134	**	0.005	0.002	**	‐0.358	0.194	n.s.
Left parsorbitalis	0.178	0.002	**	‐0.413	0.192	*	0.004	0.003	n.s.	0.266	0.278	n.s.
Left parstriangularis	0.134	0.001	**	0.145	0.144	n.s.	0.004	0.002	*	‐0.073	0.209	n.s.
Left pericalcarine	0.101	0.001	**	0.202	0.114	n.s.	0.001	0.002	n.s.	‐0.325	0.165	n.s.
Left postcentral	0.097	0.001	**	0.340	0.106	**	0.004	0.001	**	0.222	0.154	n.s.
Left posteriorcingulate	0.131	0.001	**	0.308	0.142	*	0.005	0.002	**	‐0.236	0.205	n.s.
Left precentral	0.110	0.001	**	1.223	0.122	**	0.004	0.002	*	0.181	0.177	n.s.
Left precuneus	0.111	0.001	**	0.521	0.121	**	0.003	0.002	n.s.	‐0.056	0.176	n.s.
Left rostralanteriorcingulate	0.193	0.002	**	0.470	0.205	*	‐0.005	0.003	n.s.	‐0.378	0.298	n.s.
Left rostralmiddlefrontal	0.109	0.001	**	0.153	0.122	n.s.	0.005	0.002	**	0.039	0.177	n.s.
Left superiorfrontal	0.124	0.001	**	0.505	0.137	**	0.002	0.002	n.s.	0.083	0.198	n.s.
Left superiorparietal	0.099	0.001	**	0.158	0.109	n.s.	0.004	0.001	**	0.224	0.158	n.s.
Left superiortemporal	0.129	0.001	**	0.832	0.139	**	0.004	0.002	*	‐0.123	0.201	n.s.
Left supramarginal	0.114	0.001	**	0.396	0.122	**	0.005	0.002	**	0.063	0.177	n.s.
Left frontalpole	0.241	0.002	**	‐1.236	0.266	**	0.004	0.004	n.s.	0.112	0.386	n.s.
Left temporalpole	0.268	0.003	**	‐2.010	0.301	**	0.006	0.004	n.s.	‐0.518	0.436	n.s.
Left transversetemporal	0.182	0.002	**	0.027	0.194	n.s.	‐0.001	0.003	n.s.	‐0.168	0.281	n.s.
Left insula	0.125	0.001	**	1.184	0.135	**	0.002	0.002	n.s.	‐0.700	0.195	*
Right bankssts	0.146	0.001	**	‐0.094	0.157	n.s.	0.003	0.002	n.s.	0.217	0.228	n.s.
Right caudalanteriorcingulate	0.186	0.002	**	0.936	0.198	**	‐0.008	0.003	**	‐0.105	0.288	n.s.
Right caudalmiddlefrontal	0.120	0.001	**	0.226	0.130	n.s.	0.002	0.002	n.s.	0.179	0.189	n.s.
Right cuneus	0.110	0.001	**	0.037	0.118	n.s.	0.001	0.002	n.s.	‐0.334	0.170	n.s.
Right entorhinal	0.288	0.003	**	0.122	0.310	n.s.	0.004	0.004	n.s.	‐0.746	0.449	n.s.
Right fusiform	0.114	0.001	**	0.657	0.125	**	0.001	0.002	n.s.	‐0.171	0.181	n.s.
Right inferiorparietal	0.109	0.001	**	0.390	0.120	**	0.005	0.002	**	0.233	0.174	n.s.
Right inferiortemporal	0.124	0.001	**	0.539	0.135	**	0.003	0.002	n.s.	‐0.132	0.196	n.s.
Right isthmuscingulate	0.162	0.002	**	0.401	0.172	*	‐0.002	0.002	n.s.	0.223	0.249	n.s.
Right lateraloccipital	0.101	0.001	**	0.280	0.110	*	0.005	0.001	**	0.023	0.159	n.s.
Right lateralorbitofrontal	0.129	0.001	**	‐0.174	0.144	n.s.	0.004	0.002	*	‐0.110	0.208	n.s.
Right lingual	0.102	0.001	**	0.172	0.111	n.s.	0.000	0.002	n.s.	‐0.201	0.161	n.s.
Right medialorbitofrontal	0.142	0.001	**	‐0.424	0.156	**	0.003	0.002	n.s.	‐0.201	0.227	n.s.
Right middletemporal	0.123	0.001	**	0.067	0.137	n.s.	0.006	0.002	**	0.400	0.198	n.s.
Right parahippocampal	0.207	0.002	**	0.554	0.224	*	0.005	0.003	n.s.	‐0.115	0.325	n.s.
Right paracentral	0.124	0.001	**	0.492	0.134	**	0.002	0.002	n.s.	‐0.050	0.194	n.s.
Right parsopercularis	0.131	0.001	**	0.330	0.139	*	0.001	0.002	n.s.	‐0.056	0.201	n.s.
Right parsorbitalis	0.175	0.002	**	‐0.470	0.188	*	0.002	0.003	n.s.	0.159	0.273	n.s.
Right parstriangularis	0.131	0.001	**	‐0.016	0.141	n.s.	0.002	0.002	n.s.	0.052	0.204	n.s.
Right pericalcarine	0.102	0.001	**	0.199	0.112	n.s.	0.002	0.002	n.s.	‐0.336	0.163	n.s.
Right postcentral	0.102	0.001	**	0.121	0.111	n.s.	0.002	0.002	n.s.	0.251	0.161	n.s.
Right posteriorcingulate	0.129	0.001	**	0.442	0.139	**	0.000	0.002	n.s.	‐0.014	0.202	n.s.
Right precentral	0.110	0.001	**	0.992	0.124	**	0.005	0.002	**	0.411	0.179	n.s.
Right precuneus	0.110	0.001	**	0.473	0.121	**	0.004	0.002	*	‐0.148	0.176	n.s.
Right rostralanteriorcingulate	0.185	0.002	**	0.390	0.205	n.s.	0.009	0.003	**	‐0.713	0.298	n.s.
Right rostralmiddlefrontal	0.108	0.001	**	0.084	0.120	n.s.	0.003	0.002	n.s.	‐0.162	0.174	n.s.
Right superiorfrontal	0.120	0.001	**	0.499	0.131	**	0.003	0.002	n.s.	‐0.189	0.190	n.s.
Right superiorparietal	0.099	0.001	**	0.231	0.110	*	0.003	0.002	*	0.154	0.160	n.s.
Right superiortemporal	0.127	0.001	**	0.738	0.138	**	0.005	0.002	*	0.153	0.201	n.s.
Right supramarginal	0.117	0.001	**	0.723	0.127	**	0.004	0.002	*	‐0.037	0.184	n.s.
Right frontalpole	0.236	0.002	**	‐0.642	0.255	*	0.002	0.003	n.s.	‐0.248	0.369	n.s.
Right temporalpole	0.274	0.003	**	‐2.088	0.317	**	0.007	0.004	n.s.	0.219	0.459	n.s.
Right transversetemporal	0.181	0.002	**	0.511	0.198	*	0.010	0.003	**	‐0.175	0.287	n.s.
Right insula	0.130	0.001	**	1.079	0.146	**	0.005	0.002	*	‐0.468	0.211	n.s.

* *p* < 0.05, ** *p* < 0.01, both after FDR correction.

Next, these analyses were repeated using a quadratic age model (Supplemental Tables 3A‐C). None of the subcortical or cortical surface area measures showed quadratic age by sex interaction effects in variance. Cortical thickness showed significant quadratic age by sex effects in two regions; left superior frontal cortex and right lateral orbitofrontal cortex.

### 3.4 Sex differences in anatomical correlations

Finally, we tested whether females showed greater diversity than males in anatomical correlations by comparing inter‐regional anatomical associations between males and females. Using permutation testing (B = 10000), the significance of correlation differences between males and females was assessed.

**FIGURE 6:**
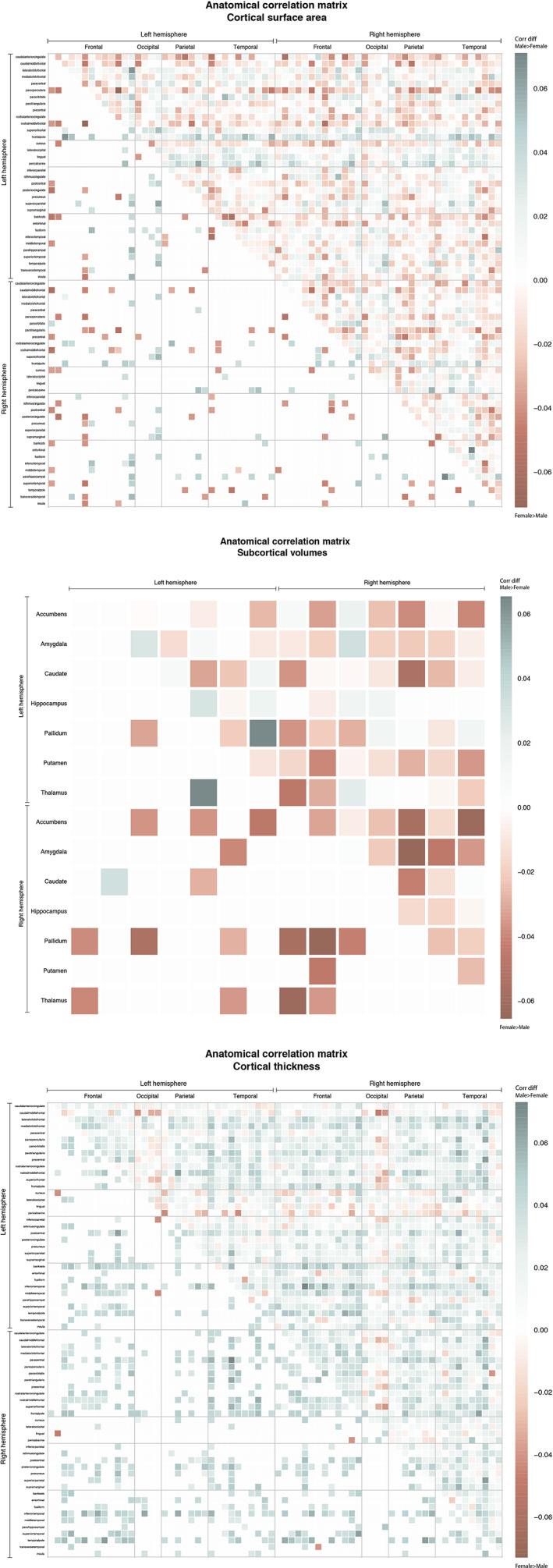
(a–c) Stronger anatomical correlations for males than females are indicated in blue (larger homogeneity in males than females), while stronger correlations for females are displayed in red (larger homogeneity in females than males). The bottom left half shows the significant variance ratio's only, using two sided permutation testing. Results are displayed for subcortical volumes (a), surface area (b), and cortical thickness (c). Cortical regions are ordered by lobe and hemisphere (left frontal, left occipital, left parietal, left temporal, right frontal, right occipital, right parietal, right temporal)

Of the 91 subcortical‐subcortical correlation coefficients, 2% showed significantly stronger correlations in males, while, unexpectedly, 19% showed stronger correlations in females (tested two‐sided) (Figure 6A). A chi‐square test of independence showed that this significantly differed between males and females, *X* ^2^ (1, *N* = 18) = 10.889, *p* < .001. For surface area, no significant difference between males and females were observed: significantly stronger male homogeneity was observed in 4% of the 2,278 unique anatomical correlations, and similarly females also showed significantly stronger correlations in 4% of the anatomical associations (Figure 6B). For thickness, stronger male than female homogeneity was observed in 21% of the correlations, while stronger female correlations were observed in <1% of the correlations (Figure 6C). This difference was significant, *X* ^2^ (1, *N* = 484) = 460.300, *p* < .001.

## 4 DISCUSSION

In this study, we analyzed a large lifespan sample of neuroimaging data from 16,683 participants spanning nine decades of life starting at birth. Results confirmed the hypothesis of greater male variability in brain structure (Forde et al. 2020; Ritchie et al. 2018; Wierenga et al. 2018, 2019). Variance differences were more pronounced for subcortical volumes and regional cortical surface area than for regional cortical thickness. We also corroborated prior findings of greater male brain structural variance at both upper and lower tails of brain measures (Wierenga et al. 2018). These variance effects seem to describe a unique aspect of sex differences in the brain that does not follow the regional pattern of mean sex differences. A novel finding was that sex differences in variance appear stable across the lifespan for around 50% of subcortical volumes, 70% of cortical surface area measures and almost all cortical thickness measures. Unexpectedly, regions with significant change in variance effects across the age range showed decreasing variance differences between the sexes with increasing age. Finally, we observed greater male inter‐regional homogeneity for cortical thickness, but not for surface area or subcortical volumes, partly replicating prior results of greater within‐subject homogeneity in the male brain (Wierenga et al. 2018). Unexpectedly, subcortical regions showed stronger interregional correlation in females than in males.

Greater male variance was most pronounced in brain regions involved in planning, regulation and inhibition of motor movements (pallidum, right inferior parietal cortex and paracentral region), episodic memory (hippocampus), and multimodal sensory integration (thalamus) (Aron, Robbins, and Poldrack 2004; Burgess, Maguire, and O'Keefe 2002; Grillner et al. 2005). In addition, the early presence of sex differences in brain structural variability may be indicative of genetic effects, in line with findings in a pediatric sample (Wierenga et al. 2018). We also observed that sex differences in structural variation are either stable or may reduce in old age. Longitudinal designs are, however, needed to address the mechanisms underlying this observation.

The expression of greater male variability in both upper and lower tails of the distribution may be related to architectural and geometric constraints that are critical for a delicate balance for effective local‐global communication. For example, neurons only partly regulate their size, and the number of neural connections does not vary strongly with neocortical size across species (Stevens 1989). Although axon size and myelin can compensate firing rates in larger brains by speeding up conduction time, there is a limited energy budget to optimize both volume and conduction time (Buzsáki, Logothetis, and Singer 2013). As such, extreme brain structure (in both directions) may come at a cost. This is in line with recent findings that show that extreme neural activity patterns may induce suboptimal expressions of mental states (Northoff and Tumati 2019). Interestingly, it has been found that individuals with autism spectrum disorder show atypical patterns of brain structure and development in both the upper and lower range (Zabihi et al. 2019), suggesting a possible link between greater male variability and vulnerability for developmental disorders (see also Alnæs et al. 2019)). Together with our findings, this opens up new approaches to understanding sex biased developmental disorders, beyond group‐level mean differences.

Although most results showed stable sex differences with increasing age, half of the subcortical regions and a quarter of the cortical surface area measures showed decreasing sex differences in variance. What stands out is that in all these regions, sex differences in variance were largest in young compared to older age. This is indicative of early mechanisms being involved. Furthermore, for subcortical regions, the patterns showed larger volumetric increases in females then in males. For surface area, interaction effects showed mostly stable variance across age in females, but decreases in variability in males. The observation that there were no significant quadratic interactions makes it unlikely that pubertal hormones may affect greater male variance. Yet, the decrease in male variance in older age, may be indicative of environmental effects later in life. Alternative explanation may be the larger number of clinical or even death rates in males that may lead to some sex difference in survival (Chen et al. 2008; Ryan et al. 1997).

Factors underlying or influencing sex differences in the brain may include sex chromosomes, sex steroids (both perinatal or pubertal), and the neural embedding of social influences during the life span (Dawson, Ashman, and Carver 2000). Although we could not directly test these mechanisms, our findings of greater male variance, that are mostly stable across age, together with the greater male inter‐regional homogeneity for cortical thickness are most in line with the single X‐chromosome expression in males compared to the mosaic pattern of X‐inactivation in females (Arnold 2012). Whereas female brain tissue shows two variants of X‐linked genes, males only show one. This mechanism may lead to increased male vulnerability, as is also seen for a number of rare X‐linked genetic mutations (Chen et al. 2008; Craig, Haworth, and Plomin 2009; Johnson, Carothers, and Deary 2009; Reinhold and Engqvist 2013; Ryan et al. 1997). None of the other sex effects mentioned above predict these specific inter and intra‐individual sex differences in brain patterns. Future studies are, however, needed to directly test these different mechanisms. Furthermore, the observation that greater male homogeneity was only observed in cortical thickness, but not cortical surface area or subcortical volumes, may speculatively indicate that X‐chromosome related genetic mechanisms may have the largest effect on cortical thickness measures.

This paper has several strengths including its sample size, the age range spanning nine decades, the inclusion of different structural measures (subcortical volumes and cortical surface area and thickness) and the investigation of variance effects. These points are important, as most observed mean sex differences in the brain are modest in size (Joel and Fausto‐Sterling 2016). We were able to analyze data from a far larger sample than those included in recent meta‐analyses of mean sex differences (Marwha et al. 2017; Ruigrok et al. 2014; Tan et al. 2016), and a very wide age range covering childhood, adolescence, adulthood and senescence. The results of this study may have important implications for studies on mean sex differences in brain structure, as analyses in such studies typically assume that group variances are equal, which the present study shows might not be tenable. This can be particularly problematic for studies with small sample sizes (Rousselet et al. 2017).

The current study has some limitations. First, the multi‐site sample was heterogeneous and specific samples were recruited in different ways, not always representative of the entire population. Furthermore, although structural measures may be quite stable across different scanners, the large number of sites may increase the variance in observed MRI measures, but this would be unlikely to be systematically biased with respect to age or sex. In addition, variance effects may change in non‐linear ways across the age‐range. This may be particularly apparent for surface area and subcortical volume measures, as these showed pronounced non‐linear developmental patterns through childhood and adolescence (Tamnes et al. 2017; Wierenga et al. 2018). Also, the imbalanced number of subjects across the age range may have diminished variability effects in the older part of the age range. The present study has a cross‐sectional design. Future studies including longitudinal data are warranted to further explore the lifespan dynamics of sex differences in variability in the brain. Last, one caveat may be the effect of movement on data quality and morphometric measures. As males have been shown to move more than females in the scanner (Pardoe, Kucharsky Hiess, and Kuzniecky 2016), this may have resulted in slight under estimations of brain volume and thickness measures for males (Reuter et al. 2015). Although quality control was conducted at each site using the standardized ENIGMA cortical and subcortical quality control protocols (http://enigma.ini.usc.edu/protocols/imaging-protocols/), which involve a combination of statistical outlier detection and visual quality checks and a similar number of males and females had partially missing data (52.4% males), we cannot exclude the possibility that in‐scanner subject movement may have affected the results. Nevertheless, we do not think this can explain our finding of greater male variance in brain morphometry measures, as this was seen at both the upper and lower ends of the distributions.

## 5 CONCLUSIONS

The present study included a large lifespan sample and robustly confirmed previous findings of greater male variance in brain structure in humans. We found greater male variance in all brain measures, including subcortical volumes and regional cortical surface area and thickness, at both the upper and the lower end of the distributions. The results have important implications for the interpretation of studies on (mean) sex differences in brain structure. Furthermore, the results of decreasing sex differences in variance across age opens a new direction for research focusing on lifespan changes in variability within sexes. Our findings of sex differences in regional brain structure being present already in childhood may suggest early genetic or gene‐environment interaction mechanisms. Further insights into the ontogeny and causes of variability differences in the brain may provide clues for understanding male biased neurodevelopmental disorders.

## COLLABORATORS

Members of the Karolinska Schizophrenia Project (KaSP) consortium: Farde L^1^, Flyckt L^1^, Engberg G^2^, Erhardt S^2^, Fatouros‐Bergman H^1^, Cervenka S^1^, Schwieler L^2^, Piehl F^3^, Agartz I^1,4,5^, Collste K^1^, Sellgren CM^2^, Victorsson P^1^, Malmqvist A^2^, Hedberg M^2^, Orhan F^2^. 1 Centre for Psychiatry Research, Department of Clinical Neuroscience, Karolinska Institutet, & Stockholm Health Care Services, Stockholm County Council, Stockholm, Sweden; 2 Department of Physiology and Pharmacology, Karolinska Institutet, Stockholm, Sweden; 3 Neuroimmunology Unit, Department of Clinical Neuroscience, Karolinska Institutet, Stockholm, Sweden; 4 NORMENT, Division of Mental Health and Addiction, Oslo University Hospital & Institute of Clinical Medicine, University of Oslo, Oslo, Norway; 5 Department of Psychiatry, Diakonhjemmet Hospital, Oslo, Norway.

## CONFLICT OF INTEREST

The authors declare the following competing interests: OAA: Speaker's honorarium from Lundbeck, Consultant of HealthLyti; PA: Received payments for consultancy to Shire/Takeda, Medic, educational/research awards from Shire/Takeda, GW Pharma, Janssen‐Cila, speaker at sponsored events for Shire, Flynn Pharma, Medic; TB: advisory or consultancy role for Lundbeck, Medice, Neurim Pharmaceuticals, Oberberg GmbH, Shire, and Infectopharm, conference support or speaker's fee by Lilly, Medice, and Shire, received royalities from Hogrefe, Kohlhammer, CIP Medien, Oxford University Press ‐ the present work is unrelated to the above grants and relationship; DB: serves as an unpaid scientific consultant for an EU‐funded neurofeedback trial that is unrelated to the present work; HB: Advisory Board, Nutricia Australi; CRKC: received partial research support from Biogen, Inc. (Boston, USA) for work unrelated to the topic of this manuscript; BF: received educational speaking fees from Medice; HJG: received travel grants and speakers honoraria from Fresenius Medical Care, Neuraxpharm, Servier and Janssen Cilag as well as research funding from Fresenius Medical Care; NJ and PMT: MPI of a research related grant from Biogen, Inc., for research unrelated to the contents of this manuscript; JK: given talks at educational events sponsored by Medic; all funds are received by King's College London and used for studies of ADHD; DM‐C: receives fees from UpToDate, Inc and Elsevier, all unrelated to the current work; AMM: received research support from Eli Lilly, Janssen, and the Sackler Foundation, and speaker fees from Illumina and Janssen; DJS: received research grants and/or honoraria from Lundbeck and Sun. The remaining authors declare no competing interests.

## AUTHOR CONTRIBUTIONS

LMW developed the theoretical framework and prepared the manuscript with support from GED, PMT, EAC, SF, and CKT. LMW designed the models and scripts, GED and SF analyzed the data. All sites processed the imaging data and conducted quality control. GD, DD, and SF brought together and organized the datasets. *Cohort PI/ENIGMA core*: DD, IA, OAA, PA, TB, AB, DIB, SB, DB, HB, GFB, DMC, XC, TMCA, CRKC, VPC, PJC, AC, DvE, SEF, BF, ADG, DCG, IHG, HJG, OG, PG, REG, RCG, LdH, BJH, PJH, OAvdH, FMH, HEHP, CH, NJ, JAJ, AJK, JK, LL, ISL, CL, NGM, DM‐C, BM, BCM, CMcD, AMM, KLM, JMM, LN, JO, PP, EP‐C, MJP, JR, JLR, PGPR, MDS, PSS, TDS, AJS, KS, AS, JWS, IES, CS‐M, AJS, DJS, SIT, JNT, DJV, HW, YW, BW, LTW, HCW, SCRW, MJW, MVZ, GIdZ, YW, PMT, EAC, SF. *Image data collection*: IA, TNA, AA‐E, KIA, PA, SB, RB‐S, AB, AB, SB, JB, AdB, AB, VDC, XC, FXC, TMCA, VPC, AC, FC, CGD, DvE, PF‐C, EJCdG, ADG, DCG, IHG, HJG, PG, REG, LdH, BH, BJH, SNH, IBH, OAvdH, IBB, CAH, DJH, SH, AJH, MH, NH, FMH, CH, ACJ, EGJ, AJK, KKK, JL, LL, LdH, ISL, CL, MWJM, BM, BCM, YW, CMcD, AMM, GM, JN, YP, PP, GP, EP‐C, JR, SS, AR, GR, JLR, PSS, RS, SS, TDS, AJS, MHS, KS, AS, LTS, PRS, AST, JNT, AU, N, HV, LW, YW, BW, WW, JDW, LTW, SCRW, DHW, YNY, MVZ, GCZ, EAC. *Image data processing/quality control*: GED, MA, TNA, AA‐E, DA, KIA, AA, NB, SB, SE, AB, JB, AdB, RMB, VDC, EJC‐R, XC, FXC, CRKC, AC, CGD, EWD, SE, DvE, JPF, PF‐C, ADG, DCG, IHG, PG, TPG, BJH, SNH, OAvdH, AJH, MH, CH, ACJ, JJ, LK, BK, JL, ISL, PHL, MWJM, SM, IM‐Z, BM, BCM, YW, GM, DvdM, JN, RS, EJC‐R, YP, JR, GR, MDS, RS, TDS, KS, AS, LTS, PRS, SIT, AST, AU, IMV, LW, YW, WW, JDW, SCRW, KW, DHW, YNY, CKT. Manuscript revision: GED, IA, MA, AA‐E, PA, AB, HB, RMB, JKB, VDC, EJC‐R, XC, AC, CGD, DD, SE, PF‐C, EJCdG, ADG, DCG, IHG, HJG, REG, RCG, TPG, BH, BJH, CAH, OAvdH, AJH, NH, FMH, ACJ, EGJ, JAJ, MK, JL, PHL, CL, DM‐C, BM, BCM, AMM, DvdM, YP, GP, EP‐C, MJP, JR, GR, PSS, RS, AJS, KS, AS, DJS, HST, AST, JNT, AU, N, HV, BW, LTW, KW, DHW.

## Supporting information


**Appendix S1:** Supplementary Information

**Supplemental Figure 1.** Boxplot visualization of comparison of Right hippocampal volume, and parahippocampal surface area and thickness before and after adjustment. As age ranges differed for each cohort adjustments were performed in two steps: initially, a linear model was used to account for cohort and non‐linear age effects. Next, random forest regression modelling was used to additionally account for field strength and FreeSurfer version. In the Left panel, volumes were not adjusted, this displays the raw data for each cohort. In the Right panel, volumes were adjusted.

**Supplemental Figure 2.** Correlation between variance ratio and vector of d‐values for each region. Results show a significant association for subcortical volumes (Left), but no significant relation for regional cortical surface area (middle), or thickness (Right).

**Supplemental Figure 3:** (A) Sex differences in variability interacted with age in 50% of the subcortical volumes. Absolute residual values are modeled across the age range. Effects showed larger male than female variance in the younger age group, and a general trend of decreasing sex differences in variance with increasing age. (B) Sex differences in variability interacted with age in 30% of cortical surface area measures. Absolute residual values are modeled across the age range. Effects showed larger male than female variance in the younger age group, and a general trend of decreasing sex differences in variance with increasing age.

**Supplementary Table 1.** Screening Process and Eligibility Criteria, Scanner, Image Acquisition Parameters and Image Segmentation Software

**Supplementary Table 2.** Supplementary Tables.

## Data Availability

The data that support the findings of this study are available on request from the corresponding author. The data are not publicly available due to privacy or ethical restrictions.
