# Supplementary material for: Greater male than female variability in regional brain structure across the lifespan
Source: Hum Brain Mapp. 2020 Oct 12;43(1):470–99. doi: 10.1002/hbm.25204 (PMC8675415; doi:10.1002/hbm.25204)
Supplement: Supplementary file 2 — Supplemental Figure 1. Boxplot visualization of comparison of Right hippocampal volume, and parahippocampal surface area and thickness before and after adjustment. As age ranges differed for each cohort adjustments were performed in two steps: initially, a linear model was used to account for cohort and non‐linear age effects. Next, random forest regression modelling was used to additionally account for field strength and FreeSurfer version. In the Left panel, volumes were not adjusted, this displays the raw data for each cohort. In the Right panel, volumes were adjusted. [file HBM-43-470-s005.pdf]

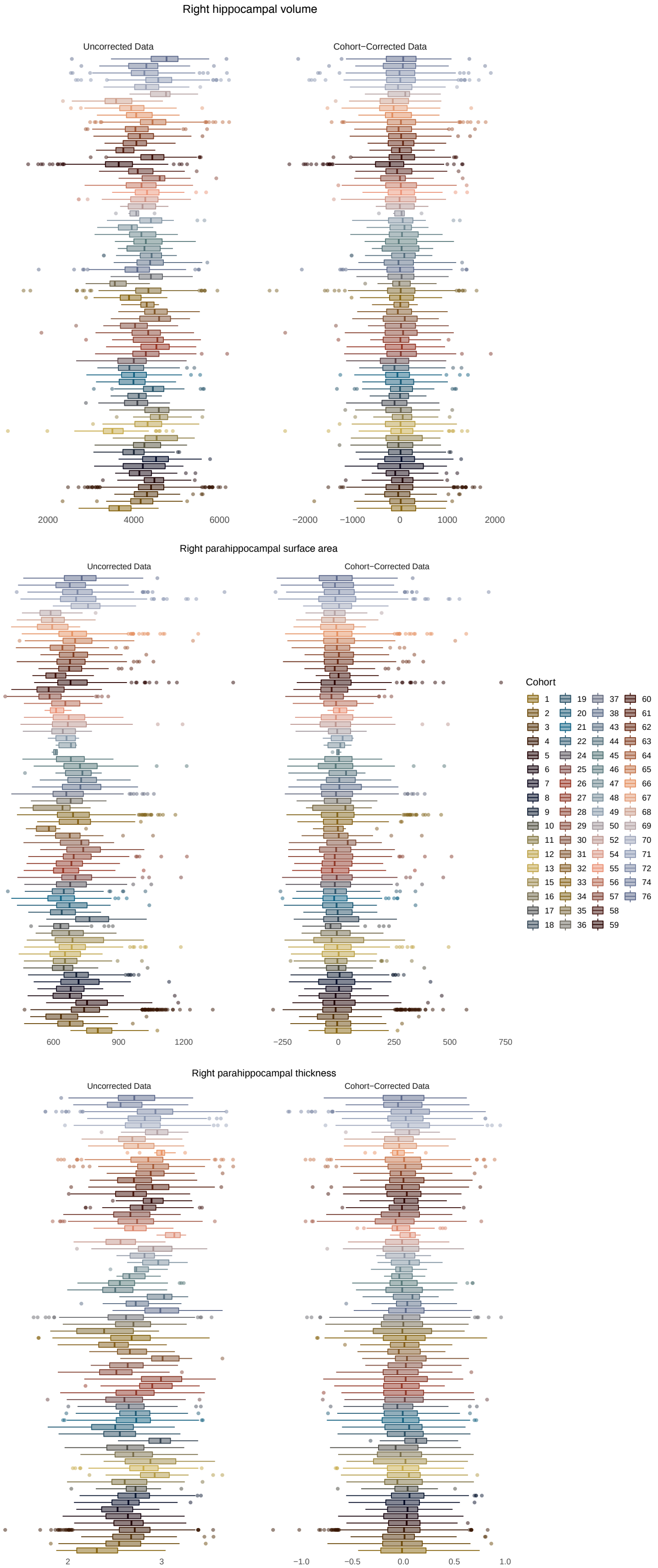

Supplemental Figure 1. Boxplot visualization of comparison of right hippocampal volume, parahippocampal surface area and thickness before and after adjustment. As age ranges differed for each cohort this was done in two steps: initially, a linear model was used to account for cohort effects and non-linear age effects, using a third degree polynomial function. Next, random forest regression modelling was used to additionally account for field strength and FreeSurfer version. In the left panel, volumes were not adjusted, this displays the raw data for each cohort. In the right panel, volumes were adjusted.
