# Supplementary figures and images for: Greater male than female variability in regional brain structure across the lifespan
Source: Hum Brain Mapp. 2020 Oct 12;43(1):470–99. doi: 10.1002/hbm.25204 (PMC8675415; doi:10.1002/hbm.25204)

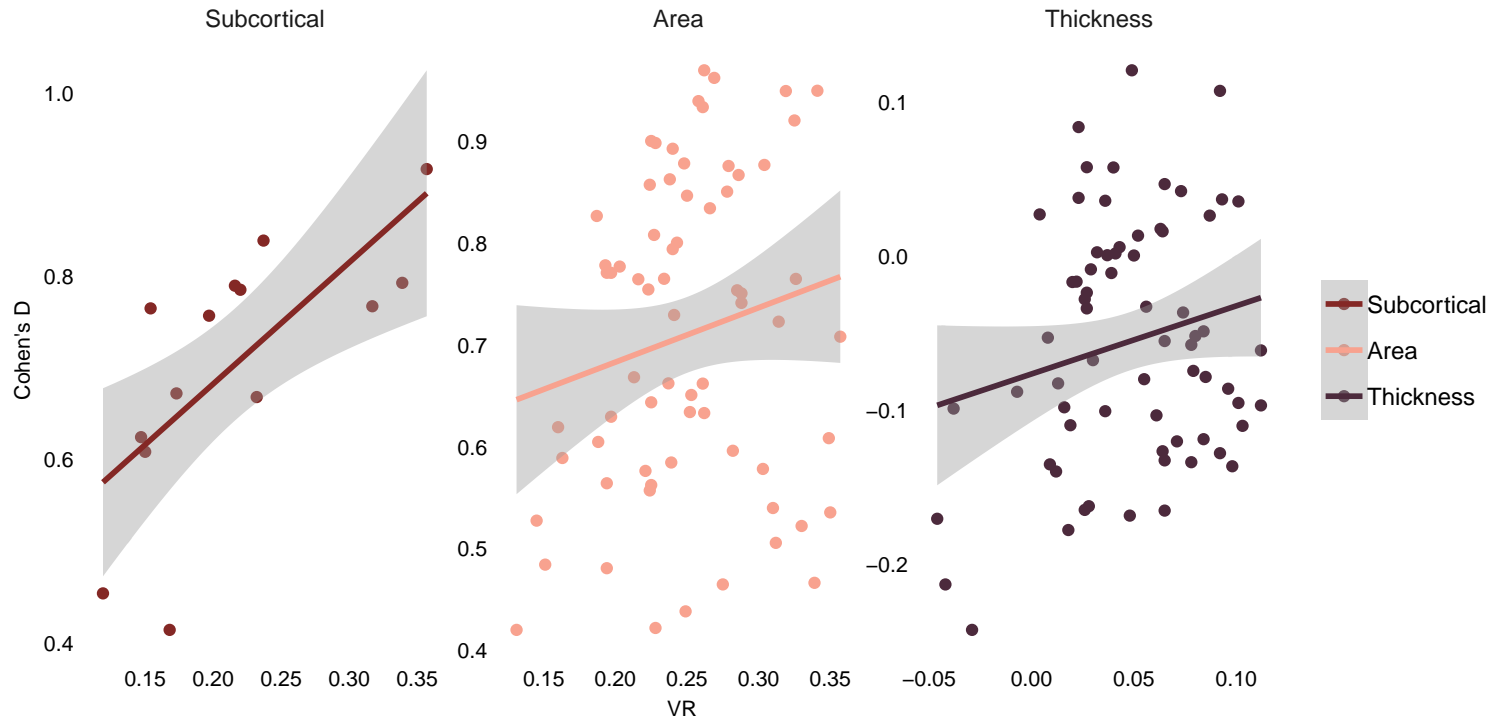

Supplement: Supplementary file 3 — Supplemental Figure 2. Correlation between variance ratio and vector of d‐values for each region. Results show a significant association for subcortical volumes (Left), but no significant relation for regional cortical surface area (middle), or thickness (Right). [file HBM-43-470-s004.pdf]
