# Supplementary material for: Greater male than female variability in regional brain structure across the lifespan
Source: Hum Brain Mapp. 2020 Oct 12;43(1):470–99. doi: 10.1002/hbm.25204 (PMC8675415; doi:10.1002/hbm.25204)
Supplement: Supplementary file 4 — Supplemental Figure 3: (A) Sex differences in variability interacted with age in 50% of the subcortical volumes. Absolute residual values are modeled across the age range. Effects showed larger male than female variance in the younger age group, and a general trend of decreasing sex differences in variance with increasing age. (B) Sex differences in variability interacted with age in 30% of cortical surface area measures. Absolute residual values are modeled across the age range. Effects showed larger male than female variance in the younger age group, and a general trend of decreasing sex differences in variance with increasing age. [file HBM-43-470-s003.pdf]

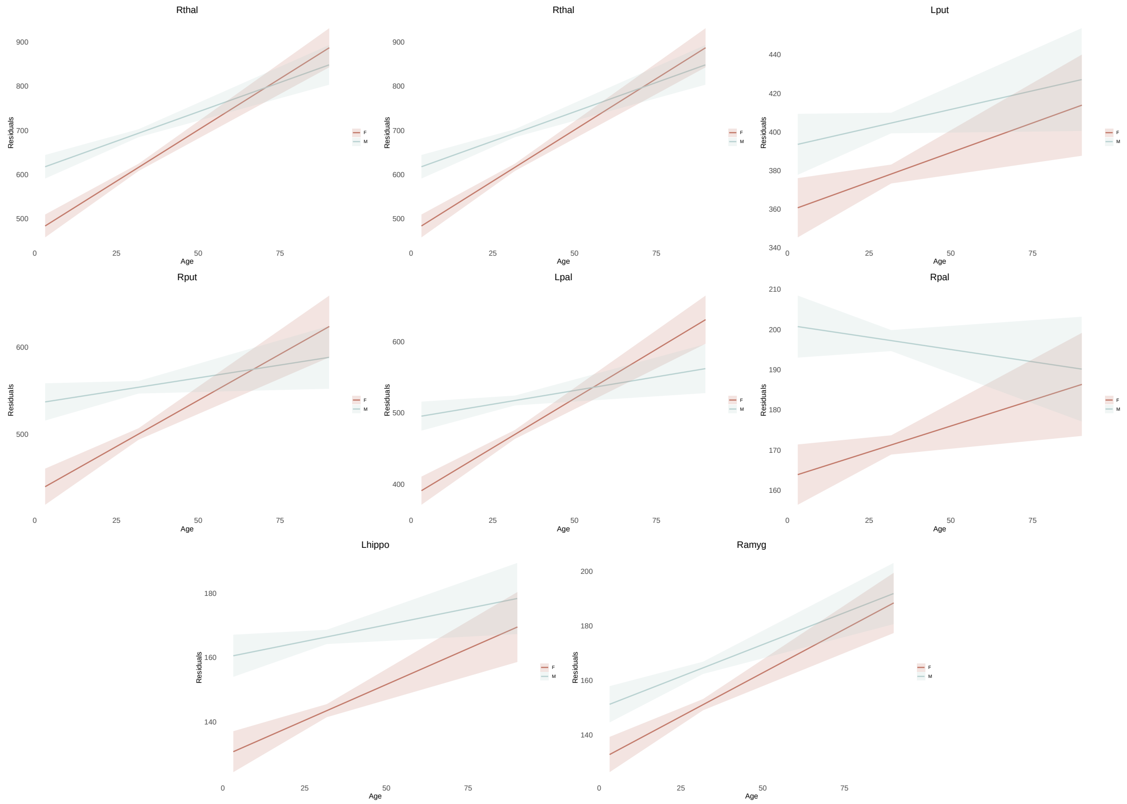

Supplemental Figure 3A. Sex differences in variability interacted with age in 50% of the subcortical volumes. Absolute residual values are modeled across the age range. Effects showed larger male than female variance in the younger age group, and a general trend of decreasing sex differences in variance with increasing age.

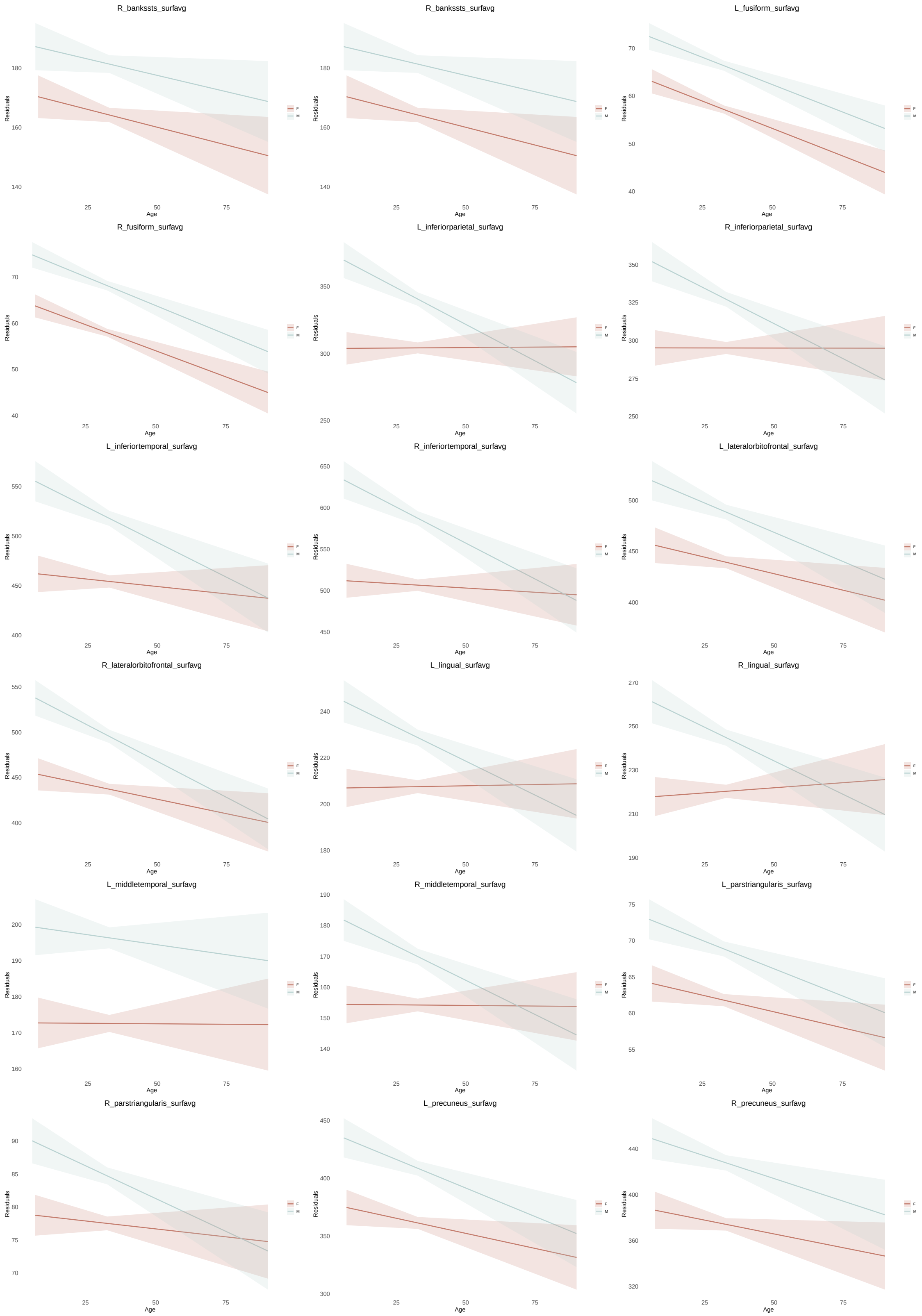

Supplemental Figure 3B-1. Sex differences in variability interacted with age in 30 % of cortical surface area measures. Absolute residual values modeled across the age range. Effects showed larger male than female variance in the younger age group, and a general trend of decreasing sex differences in variance with increasing age.

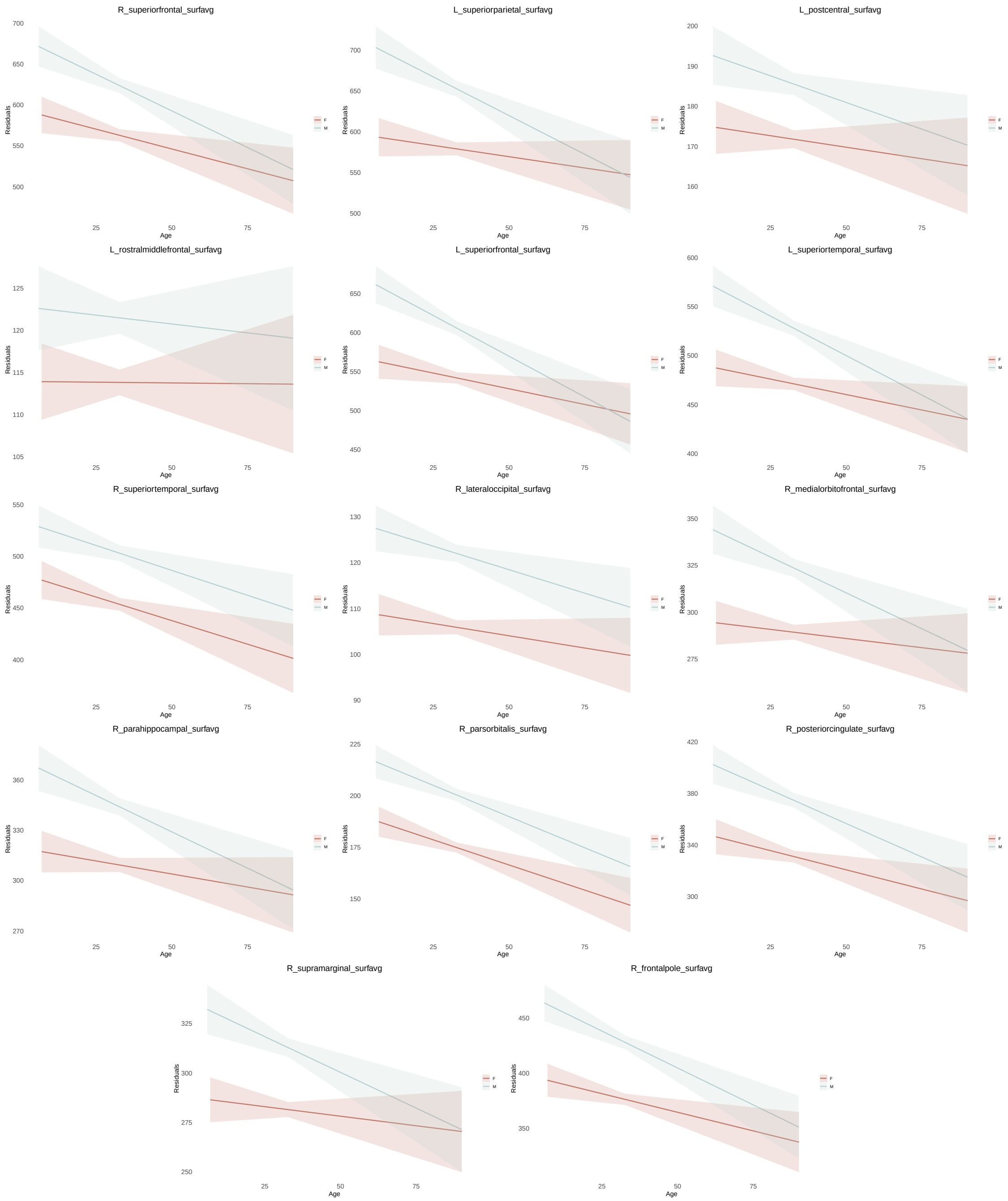

Supllemental Figure 3B-2. Sex differences in variablity interacted with age in 30% of cortical surface area measures. Absolute residual values are modeled across the age range. Effects showed larger male than female variance in the younger age group, and a general trend of decreasing sex differences in variance with increasing age.
