# Supplementary material for: Greater male than female variability in regional brain structure across the lifespan
Source: Hum Brain Mapp. 2020 Oct 12;43(1):470–99. doi: 10.1002/hbm.25204 (PMC8675415; doi:10.1002/hbm.25204)
Supplement: Supplementary file 5 — Supplementary Table 1. Screening Process and Eligibility Criteria, Scanner, Image Acquisition Parameters and Image Segmentation Software [file HBM-43-470-s006.pdf]

Supplemental Table 1. Screening Process and Eligibility Criteria, Scanner, Image Acquisition Parameters and Image Segmentation Software

Sample	Screening Process	Eligibility Criteria	Magnet strength/ Scanner Vendor	Acquisition parameters	Freesurfer version
**ADHD-NF**	KSADS	No head trauma, no neurological and psychiatric history, no lifetime alcohol or substance abuse, no previous or current use of psychotropic medication, IQ>75.	3T Siemens Tim Trio	T1-weighted 3D MPRAGE; TR/TE/TI/FA=2300ms/3030ms/900ms/9º; Image matrix= 256 × 256; 192 sagittal slices; voxel size=1mm^3	5.3
**AMC**	Personal Interview	No head trauma, no medical, neurological or psychiatric history, no lifetime alcohol or substance abuse, no previous or current use of psychotropic medication, IQ>75. No history of any psychiatric disorders in 1st degree family members.	3T Philips Intera	T1-weighted 3D MPRAGE; TE range= 3.5-4.6ms, TR range= 9-9.663ms, FA=	5.1
**Basel**	Personal Interview	No head trauma, no medical, neurological, or psychiatric history, no alcohol or substance abuse ever, no previous or current use of psychotropic medication, IQ>75. No history of any psychiatric disorders in 1st degree family members.	3T Siemens	T1-weighted MPRAGE; TR/TE/TI/FA= 2000 ms/3.37 ms/1000 ms/8º; image matrix= 256×256x160; voxel size=1x1x1 mm^3; sagittal plane	6.0
**Betula**	Personal Interview	No head trauma, no medical, neurological or psychiatric history, no lifetime alcohol or substance abuse, no previous or current use of psychotropic medication.	3T General Electric Discovery MR750	T1-weighted MPRAGE; TR/TE/TI/FA=8.1240 ms/3.2000 ms/450ms/ 12°; image matrix = 256x256	5.3
**Barcelona (1)**	KSADS	No head trauma, no medical, neurological or psychiatric history, no lifetime alcohol or substance abuse, no previous or current use of psychotropic medication, IQ>75. No history of any psychiatric disorders in 1st degree family members.	1.5 T General Electric Signa	T1-weighted; image matrix = 256 x 256; 128 slices; voxel size 1 x 1 x 1 mm^3.	5.3
**Barcelona (2)**	KSADS	No head trauma, no medical, neurological or psychiatric history, no lifetime alcohol or substance abuse, no previous or current use of psychotropic medication, IQ>75. No history of any psychiatric disorders in 1st degree family members.	3 T Siemens MAGNETOM TIM Trio	T1-weighted; image matrix = 256 x 256; 240 slices; voxel size 1 x 1 x 1 mm^3.	5.3

**BIG**	Questionnaire for psychiatric history	No head trauma, no medical, neurological or psychiatric history, no lifetime alcohol or substance abuse, no previous or current use of psychotropic medication, IQ>70. No history of any psychiatric disorders in 1st or 2nd degree family members.	1,5 T Siemens Sonata and Avanto and 3 T Siemens Trio, TimTrio and Skyra	T1-weighted 3D MPRAGE; TR/TE/TI/sagittal slices = 1940-2730 ms/850-110 ms/2.92-4.58 ms; 176-192 sagittal slices; voxel size= 1.0x1.0x1.0 mm^3	5.3
**Bonn**	Personal interview	No head trauma, no medical, neurological or psychiatric history, no previous or current use of psychotropic medication.	3T Siemens Trio	TR/TE/FA= 1570-1660ms/2.75-3.42ms/8-9°	NA
**Bordeaux**	Personal interview	No head trauma, no previous or current use of psychotropic medication, IQ>75.	3T Phillips Achieva	T1-weighted 3D TFE; TR/TE/TI/FA= 20 ms/4.6 ms/800 ms/10°; image matrix = $256x256x180mm^3$; voxel size=$1mm^3$	5.3
**BrainSCALE**	Personal interview	No head trauma, no medical, neurological or psychiatric history, no lifetime alcohol or substance abuse, no previous or current use of psychotropic medication.	1.5T Philips Achieva	T1-weighted 3D SPGR; TR/TE/ FA= 30 ms/4.6 ms/30°; image matrix=256x256; 160–180 contiguous coronal slices; voxel size=1 x 1 x 1.2 mm^3	5.1
**BRCATLAS**	Telephone interview	No head trauma, no medical, neurological or psychiatric history, no lifetime alcohol or substance abuse, no mild cognitive impairment, no previous or current use of psychotropic medication, IQ>75.	3T General Electric Signa HDx	T1 weighted: TR/TE/TI/FA= 6.9 ms/2.8 ms/650 ms/8°; image matrix = $256x256x180mm^3$; voxel size=$1mm^3$; sagittal plane acquisition	5.3
**CAMH**	SCID	No head trauma, no neurological or psychiatric history, no alcohol or substance abuse preceding 6 months, no previous or current use of psychotropic medication, IQ>75. No history of any psychotic disorders in 1st degree family members.	GE 1.5 T (echospeed)	124 Axial inversion recovery–prepared spoiled gradient recall images, 1.5-mm-thick slice acquisition TE=5.3ms; TR=12.3ms; time to inversion, 300.0ms; flip angle, 20°.	5.3
**Cardiff**	MINI	No head trauma, no medical history, including neurological and psychiatric history, no alcohol or substance abuse in the preceding 6 months, no previous or current use of psychotropic medication.	3T General Electric Signa	T1-weighted 3D FSPGR; TR/TE/TI/FA=7.9ms/3.0ms/450ms/20°; Image matrix= 256 × 192 x 172; voxel size=$1mm^3$	5.3
**CEG**	Teacher and parent Conners'	No head trauma, no medical history, including neurological and psychiatric history, no alcohol or substance abuse in the preceding 6 months, no previous or current use of psychotropic medication. IQ>75	3T General Electric Signa	T1-weighted 3D SPGR; TR/TE/ FA=2000ms/30ms/90°; Image matrix= 128 × 128; 43 slices	5.3

**CIAM**	SCID	No head trauma or psychiatric history, no previous or current use of psychotropic medication, IQ>75.	3T Siemens Allegra	T1-weighted 3D MPRAGE; TR/TE/TI/FA= 2530 ms/1.53, 3.21, 4.89, 6.57/2.91 ms/ ms/7º; image matrix= 256x256; 128 sagittal slices; voxel size= 1.3x1.0x1.3 mm^3	5.3
**CLiNG**	Personal Interview	No head trauma, no medical, neurological or psychiatric history, no lifetime alcohol or substance abuse, no previous or current use of psychotropic medication, IQ>75. No history of any psychiatric disorders in 1st degree family members.	3T Siemens Tim Trio	T1-weighted 3D MPRAGE; TR/TE/TI/FA=2250 ms/3.26 ms/900 ms/9°; image matrix = 256 x 256; 192 sagittal slices; voxel size= 1 mm^3	5.3
**CODE**	SCID Interview	No history of or current Axis-1 or 2 disorders, history of or current neurological disorder or brain injury, Serious medical condition, Severe cognitive impairment, Substance-related abuse or dependence disorder, Use of psychotropic medication, Use of central-acting medication, Pregnancy, General MRI contraindications.	3T Siemens Trio (4 Sites), 3 T Philips Achieva (1 site)	Siemens: T1 mprage, voxel size 1 mm x 1 mm x 1 mm; TR=1900 msec; TE=2.52 msec; Sample 1: 192 slices, Sample 2: 170 slices<br>Philips: T1 3D-TFE, voxel size 1 mm x 1 mm x 1 mm; TR=8.3 msec; TE=3.8 msec; 170 slices.	5.3
**COMPULS/TS EUROTRAIN**	KSADS	No head trauma, no medical, neurological or psychiatric history, no alcohol or substance abuse preceding 6 months, no previous or current use of psychotropic medication, IQ>75. No history of any psychiatric disorders in 1st or 2nd degree family members.	3T Siemens Tim Trio and Prisma	T1-weighted 3D MPRAGE; TR/TE/FA=2300 ms/2.98 ms/9°; image matrix = 256 x 256; 176 sagittal slices; voxel size= 1x1x1.2 mm^3	5.3
**ENIGMA-HIV**	MINI	No head trauma, no medical, neurological or psychiatric history, no alcohol or substance abuse preceding 6 months, no previous or current use of psychotropic medication, IQ>75. No mild cognitive impairment	3T Siemens Allegra	T1-weighted MPRAGE; TR/TE/TI/FA=2400 ms/2.38 ms/1000 ms/ 8°; 162 slices; voxel size= 1 mm^3	5.1
**ENIGMA-OCD (1)**	MINI-Plus	No head trauma, no medical, neurological or psychiatric history, no lifetime alcohol or substance abuse, no previous or current use of psychotropic medication, IQ>75. No cognitive impairment	3T Siemens Allegra	T1-weighted 3D MPRAGE; TR/TE/TI/FA=2300 ms/3.93 ms/ 1100 ms/12º; image matrix =256×240; 160 contiguous sagittal slices; voxel size=1.3 x 1 x 1 mm^3	5.3

**ENIGMA-OCD (2)**	SCID-I	No medical or psychiatric history	1.5T Siemens Sonata	T1-weighted 3D MPRAGE; TR/TE/TI/FA=2700 ms/4 ms/ 950 ms/8º; image matrix =256×192; 160 slices; voxel size= 1 mm^3	5.3
**ENIGMA-OCD (3)**	SCID-I	No medical or psychiatric history	3T General Electric Signa	T1-weighted 3D MPRAGE; image matrix =256×256; 172 slices; voxel size= 1x0.977x0.977 mm^3	5.3
**ENIGMA-OCD (4)**	Personal Interview	No head trauma, no medical, neurological or psychiatric history, no lifetime alcohol or substance abuse, no previous or current use of psychotropic medication, IQ>75.	3T Phillips Intera	T1-weighted 3D MPRAGE; TR/TE/ FA=9.69 ms/4.60 ms/8º; image matrix =256×256; 182 slices; voxel size=1 x 1 x 1.2 mm^3	5.3
**ENIGMA-OCD (5)**	SCID	No head trauma, no neurological or psychiatric history, no lifetime alcohol or substance abuse, no previous or current use of psychotropic medication, IQ>75.	1.5T General Electric Signa	T1-weighted 3D SPGR; TR/TE/FA= 14.8 ms/ 1.7 ms/ 20º; image matrix= 256 x 256 x 124; voxel size: 0.94 x 0.94 x 1.50 mm	5.3
**ENIGMA-OCD (7)**	SCID-I/NP	No head trauma, no medical, neurological or psychiatric history, no alcohol or substance abuse in the preceding 6 months, no previous or current use of psychotropic medication, IQ>70. No history of any psychiatric disorders in 1st or 2nd degree family members	1.5 T General Electric Signa	T1-weighted 3D FSPGR; TR/TE/ FA=11.8ms/4.2ms/90º; Image matrix= 256 × 256 x 130; voxel size=1.2mm^3	5.3
**FIDMAG**	Personal interview; structured interview in part of the sample	No head trauma, no medical, neurological or psychiatric history, no lifetime alcohol or substance abuse, no previous or current use of psychotropic medication, IQ>70.	1.5 T General Electric Signa	T1-weighted MPRAGE; TR/TE/FA=2000 ms/4 ms/ 9º; image matrix=512 x 512; 180 contiguous sagittal slices; voxel size=0.56 x 0.56 x 1 mm3	5.3
**GSP**	Structured phone screen and study specific self-report battery and clinical	No head trauma, no medical, neurological or psychiatric history, no lifetime alcohol or substance abuse, no current use of psychotropic medication, normal brain anatomy following brain scan.	3T Siemens Tim Trio	T1-weighted 3D multi-echo MPRAGE; TR/TE/TI/FA =2200 ms/1.54-7 ms/ 1100/7 º; voxel size=1.2x1.2x1.2 mm	4.5

	screen				
**HUBIN**	SCID-I	No head trauma, no medical, neurological or psychiatric history, no lifetime alcohol or substance abuse, no previous or current use of psychotropic medication, IQ>75. No history of any psychiatric disorders in 1st degree family members.	1.5 T General Electric Signa	T1-weighted SPGR; TR/TE/FA= 24 ms/6 ms/35 º; 124 coronal slices; voxel size 0.86 x 0.86 x 1.50 mm3.	5.3
**HMS**	Personal Interview	No head trauma, no medical, neurological or psychiatric history, no lifetime alcohol or substance abuse, no previous or current use of psychotropic medication, IQ>75. No history of any psychiatric disorders in 1st degree family members.	1.5T Siemens Magnetom Sonata	T1-weighted 3D MPRAGE; TR/TE/TI/FA=1900 ms/4.0 ms/700 ms/15°; image matrix = 256 x 256; 176 consecutive sagittal slices; voxel size=1 mm^3	5.3
**IDIVAL (1) + (2)**	CASH	No head trauma, no medical, neurological or psychiatric history, no lifetime alcohol or substance abuse, no previous or current use of psychotropic medication, IQ>75. No history of any psychiatric disorders in 1st degree family members.	3T Siemens Alegra, Phillips Achieva 1.5T General Electric Signa	T1-weighted SPGR; TR/TE/FA=24 ms/5 ms/ 5º; image matrix=256x192; T1-weighted SPGR; TR/TE/FA=3000 ms/3.9 ms/8º; image matrix=256x256; voxel size=1mm^3; sagittal plane acquisition	5.3
**IDIVAL (3)**	Personal Interview	No lifetime history of Axis I psychiatric disorders, no mild cognitive	3T Phillips Achieva	T1-weighted SPGR; TR/TE/FA=3000 ms/4.6 ms/8º; image matrix=321x312; voxel size=1mm^3; sagittal plane acquisition	5.3
**IMAGEN**	DAWBA questionnaire clinician interview	No head trauma, no medical, neurological or psychiatric history, no previous or current use of psychotropic medication. IQ>75	3T Siemens Verio and TimTrio, Philips Achieva, General Electric Signa Excite, and Signa HDx	T1-weighted 3D MPRAGE; TR/TE/TI/FA=2300ms/3030ms/900ms/9º; Image matrix= 256 × 256;192 sagittal slices; voxel size=1mm^3	5.3
**IMH**	SCID-I/NP	No head trauma, no medical history, neurological or psychiatric history, no lifetime alcohol or substance abuse, as well as no previous or current use of psychotropic medication, IQ>75. No cognitive impairment	3T Phillips Achieva	T1-weighted 3D MPRAGE; TR/TE/FA= 7.2ms/ 3.8ms/8º; image matrix=256 x 256; 180 axial slices; voxel size=0.9mm^3	5.3

**IMpACT**	SCID-I (and SCID-II)	No head trauma, no medical, neurological or psychiatric history, no alcohol or substance abuse in the preceding 6 months, no previous or current use of psychotropic medication, IQ>70. No history of any psychiatric disorders in 1st or 2nd degree family members.	1.5 T Siemens	T1-weighted 3D-MPRAGE; TR/TE/TI/FA =2730 ms/2.95 ms/1000 ms; 176 consecutive sagittal slices; voxel size= 1 mm^3	5.3
**Indiana (1)**	Personal Interview	No head trauma, no medical, neurological or psychiatric history, no lifetime alcohol or substance abuse, no previous or current use of psychotropic medication, IQ>75.	1.5T General Electric Signa Horizon LX	T1-weighted 3D SPGR; TR/TE/FA=25 ms/3 ms/ 45º; image matrix= 256 x 256; 124 contiguous coronal slices	5.1
**Indiana (2)**	Personal interview Structured phone screen	No head trauma, no medical, neurological or psychiatric history, no alcohol or substance abuse in the preceding 6 months, no previous or current use of psychotropic medication, IQ>75.	3T Siemens Magnetom TrioTim	T1-weighted MPRAGE; TR/TE/FA=2300 ms/2.95 ms/ 9º; image matrix=256 x 240; 176 contiguous sagittal slices	5.1
**Indiana (3)**	Personal interview Structured phone screen	No head trauma, no medical, neurological or psychiatric history, no alcohol or substance abuse in the preceding 6 months, no previous or current use of psychotropic medication, IQ>75.	3T Siemens Skyra	T1-weighted MPRAGE GRAPPA2; TR/TE/FA=2300 ms/2.91 ms/ 9º; image matrix=256 x 240; 160 contiguous sagittal slices	5.1
**Johns Hopkins**	Personal Interview	No head trauma, no medical, neurological or psychiatric history, no alcohol or substance abuse preceding 6 months, never prescribed with psychotropic medication, IQ>75. No mild cognitive impairment	1.5T General Electric Signa	T1-weighted SPGR; TR/TE/FA=35 ms/5 ms/ 45º; image matrix=256x256; 124 slices	5.3
**KaSP**	MINI	No head trauma, no medical, neurological or psychiatric history, no lifetime alcohol or substance abuse, no previous or current use of psychotropic medication, IQ>75. No history of any psychiatric disorders in 1st or 2nd degree family members.	3T General Electric	T1-weighted SPGR; TR/TI/FA=7.904 ms/450 ms/12 º; image matrix= 256 x 256 mm^3; 145 sagittal slices ; voxel size=0.934 x 0.934 x 1.2 mm3	5.3
**Leiden**	Self-report	No psychiatric or neurological disorders, no use of psychotropic medications	3T Philips Achieva	T1-weighted 3D SPGR; TR/TE = 9.76 ms/4.59 ms; image matrix=256x256; 160–180 contiguous coronal slices; voxel size=0.875x 0.875 x 1.2 mm^3	5.3

**MCIC (1) + (2)**	SCID, SCID-I/NP, CASH	No head trauma, no medical, neurological or psychiatric history, no lifetime alcohol or substance abuse, no previous or current use of psychotropic medication, IQ>75.	1.5T Siemens Sonata-3T Siemens Trio	T1-weighted MPRAGE sequence; TR/TE/TI/FA=2530 ms/4.76 ms/1100 ms/20°; image matrix=256×256×128 cm; voxel size=0.625 mm^3	5.3
**Meth-CT**	SCID DSM-IV	No head trauma, no medical, neurological or psychiatric history, no lifetime alcohol or substance abuse, no previous or current use of psychotropic medication, IQ>70.	3T Siemens Allegra	T1-weighted 3D MPRAGE; TR/graded TE/FA=2530 ms/ 1.53, 3.21, 4.89, 6.57 ms/ 7°; 160 contiguous sagittal slices; voxel size=1 x 1 *x 1 mm3	5.3
**MHRC**	Personal interview	No head trauma, no medical history, including neurological and psychiatric history. No Family History of neurological or psychiatric disorders	3T Philips Achieva	T1-weighted TFE; TR/TE/ FA=8.2ms/3.7ms/8°; voxel size=0.83 x 0.83 x 1 mm^3	5.3
**Muenster**	SCID	No head trauma, no medical, neurological or psychiatric history, no lifetime alcohol or substance abuse, no previous or current use of psychotropic medication, IQ>75.	3T Phillips Intera	T1 weighted TFE: TR/TE/FA= 7.4 ms/3.4 ms/9°; image matrix = 256x204x160mm^3; voxel size=0.5mm^3; sagittal plane acquisition	5.3
**NESDA**	CIDI	No lifetime history of Axis-I diagnoses, no lifetime medical or neurological morbidity including hypertension, no lifetime substance dependence, no substance abuse in the preceding year, no medication use.	3T Philips Achieva SENSE-6 to 8 channel head coil	T1-weighted 3D MPRAGE; TR/TE/FA= 9 ms/3.5 ms/8°; image matrix=256x256; 170 sagittal slices; voxel size=1mm^3	5.0
**NeuroIMAGE**	KSADS-PL	No head trauma, no mild cognitive impairment, neurological or psychiatric history, no previous or current use of psychotropic medication, IQ>75. No history of any psychiatric disorders in 1st and 2nd degree family members.	1.5 T Siemens AVANTO (Donders Centre for Cognitive Neuroimaging) 1.5 T Siemens SONATA (VU University Amsterdam)	MPRAGE 176 sagittal slices, repetition time=2,730ms, echo time=2.95ms, voxel size=1.0x1.0x1.0mm, field of view=256 mm	5.3
**Neuroventure**	DAWBA and BSI	No head trauma, no medical, neurological or psychiatric history, no lifetime alcohol or substance abuse, no previous or current use of psychotropic medication, IQ>75.	3T SIEMENS TrioTim	T1-weighted 3D MPRAGE; TR/TE/ FA= 2300 ms/2.96 ms/9°; image matrix= 256x256; voxel size= 1.0x1.0x1.0 mm^3	5.3
**NTR (1)**	MINI, BDI, STAI, STAS, YBOCS	No head trauma, no previous or current use of psychotropic medication, normal IQ.	3T Philips Intera	T1-weighted 3D MPRAGE; TR/TE/FA=9.64 ms/4.60 ms/8 ^{o}; image matrix=256 x 256; 182 coronal slices; voxel size=1 x1x1.2 mm^3	5.1

**NTR (2)**	CIDI, MADRS, BDI, STAI	No current psychiatric disorder, no current use of psychotropic medication, normal IQ.	1.5 T Siemens Sonata	T1-weighted 3D MPRAGE; TR/TE/TI/FA= 15 ms/7 ms/300 ms/8°; image matrix=256x176; 160 coronal slices; voxel size=1x1x1.5 mm^3	5.1
**NTR (3)**	DISC-IV	No head trauma, no medical, neurological or psychiatric history, no lifetime alcohol or substance abuse, no mild cognitive impairment, no previous or current use of psychotropic medication, IQ>75.	1.5T Siemens Sonata	T1-weighted 3D MPRAGE; TR/TE/TI/FA=1900 ms/3.93 ms/1100 ms/ 15°; image matrix=256 x 224; 160 sagittal slices; voxel size=1 mm^3	5.1
**NU**	SCID	No head trauma, no medical, neurological or psychiatric history, no lifetime alcohol or substance abuse, no previous or current use of psychotropic medication, IQ>75. No history of any psychiatric disorders in 1st degree family members.	1.5T SIEMENS Vision	T1-weighted 3D MPRAGE; TR/TE/TI/FA=2200 ms/4.13 ms/766 ms/13°; voxel size =0.8mm^3; axial plane acquisition.	5.3
**NUIG**	SCID	No history of head trauma or injury; No family history of ANY psychiatric disorders in 1st degree family members; IQ>75	1.5T Siemens Magnetom Symphony	T1-weighted 3D MPRAGE; TR/TE=1140/4.38 mm Image matrix = 256x256; FOV=230mm; voxel size: 0.45x0.45x0.9mm	5.1
**NYU**	SCID-NP for DSM-IV	No head trauma, no medical history, including neurological and psychiatric history, no lifetime alcohol or substance abuse, no previous or current use of psychotropic medication. IQ>75.	3T Siemens Allegra	T1-weighted 3D MPRAGE; TR/TE/TI/FA=2530ms/3.25ms/1100ms/7º	5.3
**OATS (1-4)**	Personal interview	No head trauma, no current diagnosis of a psychotic disorder, no neurological disorder, no malignancy (other than skin cancer) or other severe medical comorbidity, no mild cognitive impairment, IQ>75.	1.5T Philips Gyroscan, Siemens Magnetom Avanto, Siemens Sonata; 3T Philips Achieva Quasar Dual, a	T1-weighted 3D acquisition; TR/TE/TI/FA=15370 ms/3.24 ms/780 ms/8º; 144 slices; voxel size=1 x 1 x 1.5 mm^3	5.3
**OLIN**	SCID I	No head trauma, no medical, neurological or psychiatric history, no alcohol or substance abuse preceding 6 months, never prescribed with psychotropic medication, IQ>75.	3T Siemens Alegra	T1-weighted 3D MPRAGE; TR/TE/TI/FA= 2300 ms/2.91 ms/900 ms/9º; image matrix= 256x240x192; 160 sagittal slices; voxel size= 1.0x1.0x1.2 mm^3	5.1
**QTIM**	CIDI	No head trauma, no medical history, neurological and psychiatric history, no alcohol or substance abuse in the preceding 6	4T Bruker	T1-weighted 3D MPRAGE: TR/TE/TI/FA = 1500 ms/3.35 ms/ 700 ms/ 8°; image matrix= 256 × 256 × 256 or 256 × 256 ×	5.1

		months, no antidepressant medication or medication affecting cognition.		240; 256 coronal slices; voxel size= 0.9 mm^3	
**Oxford**	KSADS	No head trauma, no medical, neurological or psychiatric history, no lifetime alcohol or substance abuse, no previous or current use of psychotropic medication, IQ>75.	1.5T Siemens Sonata	T1-weighted 3D MPRAGE; TR/TE =12 ms/5.6 ms; image matrix =256×240x 208 mm^3; voxel size=1 mm^3	5.3
**Sao Paulo-1**	SCID	No head trauma, neurological or psychiatric history, no lifetime alcohol or substance abuse.	1.5T Siemens Espree	T1-weighted 3D MPRAGE; TR/TE/TI/FA=2400 ms/3.65 ms/ 0 ms/8º; 160 contiguous sagittal slices; voxel size=1.3 x 1.3x 1.2 mm3	5.3
**Sao Paulo-3**	SCID	No head trauma, neurological or psychiatric history, no lifetime alcohol or substance abuse. IQ>75	1.5T General Electric Signa	T1-weighted FSPGR ; TR/TE/TI/FA=21.7 ms/52 ms /20º; 124 axial slices; voxel size= 0.86 x 0.86 x 1.5 mm3	5.3
**SHIP-2**	M-CIDI interview	No head trauma, no neurological and psychiatric history, no current alcohol abuse, no current use of psychotropic medication. No cognitive impairment.	1.5T Siemens Avanto	T1-weighted 3D MPRAGE; TR/TE/ FA=1900ms/3.4ms/15º; voxel size=1mm^3	5.1 (cortical)<br>5.3 (subcortical)
**SHIP-TREND**	M-CIDI interview	No head trauma, no neurological and psychiatric history, no current alcohol abuse, no current use of psychotropic medication. No cognitive impairment.	1.5T Siemens Avanto	T1-weighted 3D MPRAGE; TR/TE/ FA=1900ms/3.4ms/15º; voxel size=1 mm^3	5.1 (cortical)<br>5.3 (subcortical)
**Stages-Dep**	SCID-I	No head trauma, no medical, neurological or psychiatric history, no lifetime alcohol or substance abuse, no previous or current use of psychotropic medication, IQ>75. No history of any psychiatric disorders in 1st degree family members.	3T Phillips Achieva	T1-weighted 3D-MPRAGE; TR/TE/TI/FA =6.7 ms/3.2 ms/200 ms/88º; °; image matrix = 288 x 288; 170 consecutive sagittal slices; voxel size= 0.896×0.896×1.2 mm^3	5.1
**Stanford**	SCID	No head trauma, no medical, neurological or psychiatric history, no lifetime alcohol or substance abuse, no previous or current use of psychotropic medication. no mild cognitive impairment.	1.5T General Electric Signa Excite	T1-weighted SPGR; TR/TE/TI/FA=8.3-10.3 ms/1.7-3.0 ms/300 ms/15º; image matrix= 256 x 192; 176 contiguous sagittal slices; voxel size=0.86x0.86x1.5 mm^3; sagittal plan acquisition	5.3

**StrokeMRI**	Personal interview	No head trauma, no medical, neurological or psychiatric history, no lifetime alcohol or substance abuse, no previous or current use of psychotropic medication, IQ>75.	3T General Electric 750 Discovery MRI scanner	T1-weighted FSPGR (BRAVO); TR/TE/TI/FA=8.16 s/3.18 ms/450 ms/12°; 188 sagittal slices; voxel size= 1.0x1.0x1.2 mm; FOV: 256 x 256 mm; scan time: 4:43 min	5.3
**SYDNEY**	SCID	No head trauma, no medical history, neurological or psychiatric history, no alcohol or substance abuse preceding 6 months, as well as no previous or current use of psychotropic medication, IQ>75.	3T General Electric Discovery MR750	T1-weighted 3D MPRAGE; TR/TE/FA= 7264ms/ 2784ms/15º; image matrix =256 x 256 x 196; voxel size=0.9mm^3	5.1
**Sydney MAS**	Personal interview	No head trauma, no diagnosis of dementia, schizophrenia, bipolar disorder no psychotic symptoms, no neurological disorder, no mild cognitive impairment, IQ>75.	3T Philips Achieva Quasar Dual	TR/TE = 6.39 ms/2.9 ms; 190 coronal slices; voxel size = 1mm^3	5.3
**TOP**	PRIME-MD	No head trauma, no organic or other psychotic disorder (ICD codes 290-299), no substance abuse in the preceding 6 months, no previous or current use of psychotropic medication, IQ>75. No history of any psychiatric disorders in 1st degree family members.	1.5T Siemens Magnetom Sonata	T1-weighted SPGR; TR/TE/TI/FA=2730 ms/3.93 ms/1000 ms/71º; voxel size = 1.33x0.94x1mm^3; sagittal plane acquisition	5.3
**Tuebingen**	SCID I and II	No head trauma, no medical history, neurological or psychiatric history, no lifetime alcohol or substance abuse as well as no previous or current use of psychotropic medication, IQ>75. No history of any psychiatric disorders in 1st degree family members.	1.5T Siemens Avanto	T1-weighted 3D MPRAGE; TR/TE/FA= 2250ms/ 3.93ms/8º; image matrix =256 x 256; voxel size=1mm^3	5.3
**UMCU**	CASH	No head trauma, no medical, neurological or psychiatric history, no lifetime alcohol or substance abuse, no previous or current use of psychotropic medication, IQ>75. No history of any psychiatric disorders in 1st degree family members.	3T Philips Achieva	T1-weighted 3D TFE; TE/TR/FA= 4.50 ms/9.96 ms/ 8˚; FOV=224mm; image matrix = 256x256 mm; voxel size=.875x.875x1 mm^3	5.3
**UNIBA**	SCID-NP	No head trauma, no medical, neurological or psychiatric history, no lifetime alcohol or substance abuse, no previous or current use	3T General Electric	T1-weighted 3D SPGR; TE/FA = min full/ 6°; image matrix= 256×256 x124	5.3

		of psychotropic medication, IQ>75. No history of any psychiatric disorders in 1st degree family members.			
**UPENN**	SCID	No head trauma, no medical history, including neurological and psychiatric history, no alcohol or substance abuse preceding 6 months, no previous or current use of psychotropic medication, IQ>75. No history of any psychiatric disorders in 1st degree family members.	3T Siemens Tim Trio	T1-weighted 3D MPRAGE; TR/TE/TI/FA=1810 ms/3.51 ms/1100 ms/9º; image matrix= 256 × 192;160 axial slices	5.3
**Yale**	KSADS-PL	No head trauma, neurological or psychiatric history, no alcohol or substance abuse in the preceding 6 months, no previous or current use of psychotropic medication, IQ>75.	3T General Electric Signa	T1-weighted 3D MPRAGE; image matrix =256×256; voxel size=0.976 x 0.976 x 1 mm3	5.3

Abbreviations of Terms: BDI = Behavioural Descriptive Interview; BSI = Brief Symptom Inventory; CASH = Comprehensive assessment of symptoms and history; CDR = Clinical Dementia Rating; CIDI = Composite International Diagnostic Interview; DAWBA = Development and Well-Being Assessment; DISC-IV = Diagnostic Interview Schedule for Children; DSM = Diagnostic and Statistical Manual of Mental Disorders (DSM); FA=flip angle; FSPGR=fast spoiled gradient echo sequence; GRE=spoiled gradient echo sequence; ICD= International Classification of Diseases; IR= inversion recovery; KSADS-PL= Kiddie Schedule for Affective Disorders and Schizophrenia-Present and Lifetime; MADRS = Montgomery-Asberg Depression Rating Scale; MINI = Mini International Neuropsychiatric Interview; MMSE = Mini Mental State Exam; PRIME-MD = Primary Care Evaluation of Mental Disorder; SCID = Structured Clinical Interview for DSM Disorders; SCID-I/NP = SCID Non-Patient version; SPGR=spoiled gradient recalled sequence; STAI = State-Trait Anxiety Inventory; STAS = State-trait anger scale; TE=echo time; TI=inversion time; TR=repetition time; TFE=turbo field echo sequence; YBOCS = Yale-Brown Obsessive Compulsive Scale

Abbreviations of studies: **ADHD-NF = Attention Deficit Hyperactivity Disorder- Neurofeedback Study; AMC = Amsterdam Medisch Centrum; Basel = University of Basel**; **Betula = Swedish longitudinal study on aging, memory, and dementia**; **BIG = Brain Imaging Genetics; Bonn = University of Bonn; Bordeaux = University of Bordeaux; BrainSCALE=Brain Structure and Cognition: an Adolescence Longitudinal twin study into Etiology; BRCATLAS= NIHR Biomedical Research Centre/ Mapping the relationship between the white matter and executive function across the adult lifespan; CAMH = Centre for Addiction and Mental Health; Cardiff = Cardiff University; CEG = Cognitive-experimental and Genetic study of ADHD and Control Sibling Pairs; CIAM = Cortical Inhibition and Attentional Modulation study; CLiNG = Clinical Neuroscience Göttingen**; **CODE = Chronic Depression Study; ENIGMA-HIV = Enhancing NeuroImaging Genetics through Meta-Analysis-Human Immunodeficiency Virus Working Group; ENIGMA-OCD = Enhancing NeuroImaging Genetics through Meta-Analysis- Obsessive Compulsive Disorder Working Group; FBIRN = Function Biomedical Informatics Research Network; EDINBURGH = The University of Edinburgh**; **GSP = Brain Genomics Superstruct Project (http://neuroinformatics.harvard.edu/gsp/; Holmes et al., Sci Data 2015; http://www.nature.com/articles/sdata201531); HMS = Homburg Multidiagnosis Study**; **ENIGMA-OCD (IDIBELL)**; **FIDMAG = Fundación para la Investigación y Docencia Maria Angustias Giménez**; **HUBIN = Human Brain Informatics; IDIVAL = Valdecilla Biomedical Research Institute; IMAGEN = the IMAGEN Consortium; IMH=Institute of Mental Health, Singapore; IMpACT = The International Multicentre persistent ADHD Genetics Collaboration; Indiana = Indiana University School of Medicine; KaSP= The Karolinska Schizophrenia Project; Leiden = Leiden University; MCIC = MIND Clinical Imaging Consortium formed by the Mental Illness and Neuroscience Discovery (MIND) Institute now the Mind Research Network (MRN; http//ww.mrn.org); Melbourne = University of Melbourne**; **Meth-CT = methamphetamine use; University of Cape Town; MHRC = Mental Health Research Center; Muenster = Muenster University; NESDA = The Netherlands Study of Depression and Anxiety**; Neuroventure = ; **NTR = Netherlands Twin Register**; **NU = Northwestern University; NYU = New York University**; **OLIN = Olin Neuropsychiatric Research Center; NUIG = National University of Ireland Galway**; **OATS = Older Australian Twins Study**; **Oxford =Oxford University; QTIM = Queensland Twin Imaging; Sao Paulo = University of Sao Paulo; SHIP = Study of Health in Pomerania; Stages-Dep= Stages of Depression Study; Stanford = Stanford University**; **StrokeMRI = Stroke Magnetic Resonance Imaging; SYDNEY = University of Sydney; Sydney MAS = Sydney Memory and Ageing Study; TOP = Tematisk Område Psykoser (Thematically Organized Psychosis Research); Tuebingen = University of Tuebingen; UMCU = Universitair Medisch Centrum Utrecht**; **UNIBA = University of Bari Aldo Moro**; **UPENN=University of Pennsylvania; Yale = Yale University**
