# Supplementary material for: Greater male than female variability in regional brain structure across the lifespan
Source: Hum Brain Mapp. 2020 Oct 12;43(1):470–99. doi: 10.1002/hbm.25204 (PMC8675415; doi:10.1002/hbm.25204)
Supplement: Supplementary file 6 — Supplementary Table 2. Supplementary Tables. [file HBM-43-470-s002.pdf]

Supplementary Table 2A

Subcortical volume	Female (n=7141)	Male (n=6555)	Mean difference test		Variance Ratio test	
	M	M	P	Cohen's D	VR	P
left thal	-174.193	189.936	**	0.504	0.233	**
right thal	-175.412	191.140	**	0.576	0.364	**
left caud	-65.407	71.566	**	0.311	0.163	**
right caud	-70.631	78.224	**	0.328	0.133	**
left put	-133.575	144.498	**	0.453	0.205	**
right put	-132.828	144.790	**	0.483	0.218	**
left pal	-54.597	59.591	**	0.510	0.321	**
right pal	-44.439	48.627	**	0.504	0.357	**
left hippo	-73.545	79.192	**	0.387	0.119	**
right hippo	-67.811	74.070	**	0.368	0.221	**
left amyg	-45.046	48.757	**	0.492	0.156	**
right amyg	-49.629	53.719	**	0.515	0.216	**
left accumb	-10.863	11.819	**	0.208	0.162	**
right accumb	-11.997	13.134	**	0.248	0.133	**

Supplementary Table 2B

Surface area	Female (n=6243)	Male (n=5092)	Mean difference test		Variance Ratio test	
	M	M	P	Cohen's D	VR	P
left bankssts	-21.428	26.514	**	0.303	0.288	**
left caudalanteriorcingulate	-7.761	9.655	**	0.138	0.144	**
left caudalmiddlefrontal	-32.171	40.016	**	0.218	0.174	**
left cuneus	-23.913	28.961	**	0.288	0.213	**
left entorhinal	-9.573	11.775	**	0.282	0.318	**
left fusiform	-59.732	73.637	**	0.401	0.218	**
left inferiorparietal	-89.239	110.767	**	0.388	0.290	**
left inferiortemporal	-69.130	84.488	**	0.403	0.198	**
left isthmuscingulate	-26.185	32.319	**	0.424	0.309	**
left lateraloccipital	-116.996	144.255	**	0.534	0.241	**
left lateralorbitofrontal	-33.519	41.514	**	0.348	0.170	**
left lingual	-44.627	54.474	**	0.276	0.210	**
left medialorbitofrontal	-32.826	40.488	**	0.373	0.276	**
left middletemporal	-55.369	68.345	**	0.396	0.261	**
left parahippocampal	-10.153	12.606	**	0.234	0.324	**
left paracentral	-19.674	24.133	**	0.270	0.294	**
left parsopercularis	-23.321	28.224	**	0.214	0.365	**
left parsorbitalis	-12.910	15.872	**	0.401	0.195	**
left parstriangularis	-26.699	32.436	**	0.326	0.267	**
left pericalcarine	-18.170	22.274	**	0.198	0.139	**
left postcentral	-75.090	92.913	**	0.462	0.314	**
left posteriorcingulate	-21.743	26.882	**	0.317	0.254	**
left precentral	-97.158	120.713	**	0.551	0.316	**
left precuneus	-67.764	83.523	**	0.437	0.283	**
left rostralanteriorcingulate	-14.519	17.886	**	0.256	0.169	**
left rostralmiddlefrontal	-136.648	169.107	**	0.549	0.293	**
left superiorfrontal	-141.866	175.228	**	0.559	0.228	**
left superiorparietal	-79.266	98.252	**	0.344	0.227	**
left superiortemporal	-85.038	104.778	**	0.592	0.208	**
left supramarginal	-94.686	117.258	**	0.487	0.304	**
left frontalpole	-3.241	3.968	**	0.221	0.231	**
left temporalpole	-8.189	10.093	**	0.316	0.219	**
left transversetemporal	-8.304	10.195	**	0.268	0.217	**
left insula	-39.534	48.789	**	0.474	0.229	**
right bankssts	-19.472	24.007	**	0.337	0.269	**
right caudalanteriorcingulate	-12.282	15.246	**	0.193	0.283	**
right caudalmiddlefrontal	-31.934	39.450	**	0.213	0.229	**
right cuneus	-26.831	32.861	**	0.330	0.236	**
right entorhinal	-9.061	11.120	**	0.260	0.331	**
right fusiform	-75.126	91.735	**	0.525	0.197	**
right inferiorparietal	-135.284	166.645	**	0.540	0.306	**
right inferiortemporal	-64.768	79.764	**	0.413	0.188	**
right isthmuscingulate	-21.334	26.370	**	0.375	0.321	**
right lateraloccipital	-114.231	139.966	**	0.515	0.267	**
right lateralorbitofrontal	-36.896	46.038	**	0.349	0.221	**
right lingual	-44.954	55.584	**	0.300	0.245	**
right medialorbitofrontal	-30.524	37.532	**	0.404	0.223	**
right middletemporal	-66.294	81.941	**	0.463	0.234	**
right parahippocampal	-14.183	17.365	**	0.367	0.319	**
right paracentral	-22.852	28.213	**	0.271	0.332	**
right parsopercularis	-20.480	25.363	**	0.211	0.323	**
right parsorbitalis	-16.572	20.550	**	0.414	0.178	**
right parstriangularis	-34.599	42.647	**	0.341	0.270	**
right pericalcarine	-19.021	23.521	**	0.198	0.151	**
right postcentral	-70.424	87.713	**	0.443	0.292	**
right posteriorcingulate	-20.435	24.929	**	0.295	0.240	**
right precentral	-110.167	136.382	**	0.587	0.339	**
right precuneus	-84.731	104.341	**	0.501	0.255	**
right rostralanteriorcingulate	-13.300	16.469	**	0.257	0.203	**
right rostralmiddlefrontal	-124.963	153.957	**	0.488	0.225	**
right superiorfrontal	-141.040	173.327	**	0.543	0.270	**
right superiorparietal	-85.848	104.679	**	0.382	0.212	**
right superiortemporal	-52.348	64.673	**	0.408	0.231	**
right supramarginal	-68.219	83.861	**	0.362	0.287	**
right frontalpole	-4.746	5.920	**	0.247	0.186	**
right temporalpole	-5.293	6.502	**	0.208	0.240	**
right transversetemporal	-5.637	6.915	**	0.245	0.178	**
right insula	-48.677	60.048	**	0.513	0.224	**

Supplementary Table 2C

Thickness	Female (n=6620)	Male (n=5913)	Mean difference test		Variance Ratio test	
	M	M	P	Cohen's D	VR	P
left bankssts	-0.001	0.002	n.s.	0.014	0.023	**
left caudalanteriorcingulate	0.024	-0.026	**	0.203	-0.035	n.s.
left caudalmiddlefrontal	0.005	-0.006	**	0.084	0.062	n.s.
left cuneus	-0.001	0.002	n.s.	0.028	0.081	*
left entorhinal	-0.016	0.018	**	0.100	0.011	n.s.
left fusiform	-0.001	0.001	n.s.	0.015	0.016	n.s.
left inferiorparietal	0.006	-0.006	**	0.115	0.126	**
left inferiortemporal	-0.004	0.005	**	0.067	-0.040	n.s.
left isthmuscingulate	0.007	-0.007	**	0.072	-0.028	**
left lateraloccipital	0.002	-0.002	*	0.044	0.111	**
left lateralorbitofrontal	-0.004	0.006	**	0.071	0.082	**
left lingual	-0.005	0.006	**	0.090	0.051	n.s.
left medialorbitofrontal	-0.006	0.008	**	0.087	0.000	n.s.
left middletemporal	-0.005	0.007	**	0.087	0.045	n.s.
left parahippocampal	0.013	-0.013	**	0.085	0.011	n.s.
left paracentral	0.004	-0.003	*	0.048	0.041	**
left parsopercularis	-0.004	0.005	**	0.066	0.063	**
left parsorbitalis	0.010	-0.011	**	0.102	0.037	**
left parstriangularis	0.002	-0.002	n.s.	0.025	0.027	**
left pericalcarine	-0.001	0.002	n.s.	0.026	0.068	**
left postcentral	0.006	-0.007	**	0.122	0.055	**
left posteriorcingulate	0.003	-0.003	n.s.	0.037	0.063	**
left precentral	0.004	-0.004	**	0.066	0.077	**
left precuneus	-0.002	0.003	n.s.	0.040	0.058	**
left rostralanteriorcingulate	0.018	-0.020	**	0.166	-0.066	n.s.
left rostralmiddlefrontal	0.002	-0.002	n.s.	0.031	0.073	**
left superiorfrontal	0.010	-0.011	**	0.174	0.037	n.s.
left superiorparietal	0.006	-0.006	**	0.119	0.138	**
left superiortemporal	-0.004	0.005	**	0.062	0.042	**
left supramarginal	0.007	-0.007	**	0.119	0.067	**
left frontalpole	0.013	-0.014	**	0.091	0.024	n.s.
left temporalpole	0.000	-0.001	n.s.	0.003	0.011	n.s.
left transversetemporal	0.017	-0.018	**	0.169	0.011	n.s.
left insula	-0.011	0.012	**	0.163	0.042	n.s.
right bankssts	-0.003	0.004	*	0.046	0.052	**
right caudalanteriorcingulate	0.025	-0.028	**	0.233	-0.061	n.s.
right caudalmiddlefrontal	0.006	-0.006	**	0.090	0.011	**
right cuneus	0.000	0.000	n.s.	0.001	0.045	*
right entorhinal	0.002	-0.003	n.s.	0.013	0.010	n.s.
right fusiform	-0.001	0.002	n.s.	0.027	0.005	n.s.
right inferiorparietal	0.005	-0.005	**	0.098	0.109	**
right inferiortemporal	-0.002	0.003	n.s.	0.035	0.002	n.s.
right isthmuscingulate	0.008	-0.009	**	0.088	-0.045	**
right lateraloccipital	0.002	-0.001	n.s.	0.022	0.098	**
right lateralorbitofrontal	0.001	0.000	n.s.	0.010	0.049	**
right lingual	-0.004	0.005	**	0.070	0.061	n.s.
right medialorbitofrontal	0.001	0.000	n.s.	0.009	0.037	n.s.
right middletemporal	-0.006	0.007	**	0.099	0.060	**
right parahippocampal	0.019	-0.020	**	0.153	0.032	n.s.
right paracentral	0.002	-0.002	n.s.	0.028	0.051	**
right parsopercularis	-0.002	0.003	n.s.	0.031	0.000	**
right parsorbitalis	0.015	-0.016	**	0.155	0.007	n.s.
right parstriangularis	0.002	-0.002	n.s.	0.029	-0.016	**
right pericalcarine	0.000	0.000	n.s.	0.002	0.028	n.s.
right postcentral	0.007	-0.007	**	0.121	-0.012	**
right posteriorcingulate	0.005	-0.005	**	0.067	-0.021	*
right precentral	0.005	-0.005	**	0.091	0.052	**
right precuneus	-0.003	0.004	**	0.059	0.048	**
right rostralanteriorcingulate	0.008	-0.008	**	0.067	0.033	n.s.
right rostralmiddlefrontal	0.003	-0.003	**	0.053	0.073	**
right superiorfrontal	0.010	-0.011	**	0.169	0.045	n.s.
right superiorparietal	0.006	-0.006	**	0.119	0.102	**
right superiortemporal	-0.006	0.007	**	0.094	0.077	**
right supramarginal	0.004	-0.004	**	0.069	0.051	**
right frontalpole	0.018	-0.019	**	0.131	-0.009	n.s.
right temporalpole	-0.010	0.011	**	0.060	0.005	n.s.
right transversetemporal	0.008	-0.008	**	0.077	0.104	*
right insula	-0.010	0.012	**	0.142	0.069	**

Supplementary Table 3A

Subcortical	Intercept	(s.e.)	P	Age2	(s.e.)	P	Sex	(s.e.)	P	Sex by age2	(s.e.)	P
left thal	613.049	6.008	**	8695.484	716.539	**	74.940	8.682	**	-1187.243	1014.991	n.s.
right thal	531.871	5.364	**	4171.752	639.774	**	95.209	7.752	**	-1244.554	906.251	n.s.
left caud	364.783	3.521	**	2190.319	419.998	**	29.834	5.089	**	834.754	594.934	n.s.
right caud	377.897	3.603	**	3303.418	429.729	**	28.284	5.207	**	479.254	608.719	n.s.
left put	495.407	4.834	**	6160.964	576.548	**	57.544	6.986	**	-323.645	816.691	n.s.
right put	464.079	4.595	**	6698.088	547.991	**	50.995	6.640	**	-614.940	776.239	n.s.
left pal	170.576	1.763	**	1939.804	210.284	**	27.772	2.548	**	-317.629	297.871	n.s.
right pal	143.455	1.497	**	1092.346	178.526	**	23.127	2.163	**	90.990	252.886	n.s.
left hippo	320.138	3.201	**	3512.741	381.787	**	31.862	4.626	**	-1374.551	540.807	n.s.
right hippo	311.867	3.121	**	2672.383	372.203	**	39.214	4.510	**	-605.863	527.232	n.s.
left amyg	151.438	1.523	**	1726.265	181.644	**	14.425	2.201	**	-576.511	257.302	n.s.
right amyg	156.565	1.596	**	1835.945	190.409	**	16.531	2.307	**	-660.039	269.717	n.s.
left accumb	85.209	0.858	**	1188.991	102.329	**	6.094	1.240	**	-58.930	144.951	n.s.
right accumb	80.431	0.786	**	1059.372	93.692	**	5.613	1.135	**	-244.057	132.716	n.s.

Supplementary Table 3B

Surface area	Intercept	(s.e.)	P	Age2	(s.e.)	P	Sex	(s.e.)	P	Sex by age2	(s.e.)	P
left bankssts	126.857	1.375	**	468.035	148.508	**	16.754	2.055	**	-144.283	217.605	n.s.
left caudalanteriorcingulate	126.787	1.375	**	439.050	148.445	**	16.823	2.054	**	-122.407	217.513	n.s.
left caudalmiddlefrontal	104.429	1.114	**	-125.449	120.351	n.s.	3.901	1.666	*	-127.352	176.348	n.s.
left cuneus	293.437	2.943	**	502.730	317.810	n.s.	22.109	4.398	**	63.451	465.680	n.s.
left entorhinal	154.473	1.609	**	-258.284	173.761	n.s.	13.102	2.405	**	285.420	254.608	n.s.
left fusiform	57.088	0.651	**	246.897	70.291	**	9.333	0.973	**	-60.093	102.996	n.s.
left inferiorparietal	303.903	3.104	**	733.041	335.160	*	36.622	4.638	**	-711.684	491.103	n.s.
left inferiortemporal	453.623	4.702	**	1832.218	507.743	**	64.389	7.027	**	-1264.030	743.985	n.s.
left isthmuscingulate	351.679	3.537	**	2118.970	381.998	**	33.152	5.287	**	-1028.874	559.733	n.s.
left lateraloccipital	116.786	1.250	**	1.485	135.027	n.s.	19.631	1.869	**	208.057	197.853	n.s.
left lateralorbitofrontal	439.073	4.476	**	408.166	483.345	n.s.	49.659	6.689	**	303.046	708.234	n.s.
left lingual	207.272	2.114	**	837.294	228.332	**	21.655	3.160	**	-397.922	334.570	n.s.
left medialorbitofrontal	310.562	3.142	**	62.805	339.266	n.s.	29.594	4.695	**	-899.943	497.120	n.s.
left middletemporal	172.650	1.796	**	-186.564	193.995	n.s.	23.477	2.685	**	-138.893	284.257	n.s.
left parahippocampal	296.470	2.997	**	1349.148	323.622	**	32.342	4.479	**	-635.081	474.196	n.s.
left paracentral	72.659	0.887	**	146.999	95.780	n.s.	10.826	1.326	**	-99.995	140.344	n.s.
left parsopercularis	133.455	1.420	**	-209.260	153.336	n.s.	18.981	2.122	**	191.968	224.681	n.s.
left parsorbitalis	192.892	2.113	**	240.095	228.190	n.s.	32.407	3.158	**	-19.421	334.363	n.s.
left parstriangularis	61.740	0.641	**	174.928	69.257	*	7.148	0.958	**	-111.706	101.481	n.s.
left pericalcarine	148.648	1.525	**	286.425	164.734	n.s.	19.352	2.280	**	-18.396	241.381	n.s.
left postcentral	171.902	1.691	**	-361.951	182.645	*	13.425	2.528	**	-136.405	267.625	n.s.
left posteriorcingulate	340.826	3.576	**	88.942	386.228	n.s.	46.216	5.345	**	-999.934	565.932	n.s.
left precentral	130.418	1.364	**	-272.664	147.314	n.s.	13.878	2.039	**	25.470	215.856	n.s.
left precuneus	361.433	3.930	**	-417.038	424.423	n.s.	46.837	5.874	**	-250.550	621.898	n.s.
left rostralanteriorcingulate	329.071	3.385	**	150.942	365.610	n.s.	44.972	5.060	**	256.184	535.721	n.s.
left rostralmiddlefrontal	113.811	1.158	**	3.816	125.011	n.s.	7.655	1.730	**	15.972	183.176	n.s.
left superiorfrontal	541.177	5.556	**	1927.368	600.057	**	65.054	8.304	**	-680.175	879.250	n.s.
left superiorparietal	578.286	6.021	**	1700.014	650.221	**	74.321	8.998	**	-1311.611	952.755	n.s.
left superiortemporal	470.859	4.787	**	931.025	516.942	n.s.	57.562	7.154	**	331.895	757.464	n.s.
left supramarginal	307.760	3.214	**	819.335	347.036	*	40.690	4.803	**	123.558	508.504	n.s.
left frontalpole	391.464	4.081	**	966.018	440.730	*	58.784	6.099	**	-331.182	645.792	n.s.
left temporalpole	25.400	0.265	**	47.772	28.616	n.s.	3.224	0.396	**	-83.854	41.930	n.s.
left transversetemporal	45.368	0.479	**	194.716	51.710	**	5.206	0.716	**	-137.908	75.770	n.s.
left insula	56.947	0.594	**	-7.686	64.171	n.s.	6.788	0.888	**	-6.444	94.028	n.s.
right bankssts	164.356	1.842	**	-399.121	198.942	*	16.907	2.753	**	164.981	291.506	n.s.
right caudalanteriorcingulate	107.054	1.139	**	374.190	122.986	**	13.963	1.702	**	-141.376	180.209	n.s.
right caudalmiddlefrontal	114.635	1.199	**	-319.007	129.509	*	14.718	1.792	**	67.915	189.767	n.s.
right cuneus	288.838	2.933	**	364.976	316.703	n.s.	30.420	4.383	**	-269.229	464.058	n.s.
right entorhinal	152.670	1.657	**	-4.937	178.965	n.s.	16.423	2.477	**	430.922	262.233	n.s.
right fusiform	57.903	0.641	**	149.574	69.216	*	10.349	0.958	**	60.903	101.421	n.s.
right inferiorparietal	294.737	2.995	**	1058.200	323.467	**	32.896	4.476	**	100.632	473.969	n.s.
right inferiortemporal	506.192	5.248	**	1296.520	566.710	*	81.216	7.843	**	-1318.390	830.387	n.s.
right isthmuscingulate	326.198	3.322	**	1526.453	358.727	**	29.511	4.964	**	-78.652	525.635	n.s.
right lateraloccipital	105.916	1.158	**	-81.030	125.054	n.s.	16.020	1.731	**	-82.175	183.240	n.s.
right lateralorbitofrontal	437.115	4.538	**	27.875	490.026	n.s.	57.833	6.782	**	-959.819	718.025	n.s.
right lingual	220.136	2.285	**	585.537	246.770	*	24.988	3.415	**	106.253	361.586	n.s.
right medialorbitofrontal	289.218	3.000	**	70.565	323.929	n.s.	34.006	4.483	**	-705.531	474.646	n.s.
right middletemporal	154.044	1.565	**	530.659	169.031	**	15.954	2.339	**	-334.799	247.678	n.s.
right parahippocampal	308.652	3.167	**	1826.197	342.056	**	35.701	4.734	**	-843.985	501.208	n.s.
right paracentral	69.859	0.780	**	147.856	84.229	n.s.	12.062	1.166	**	-6.834	123.418	n.s.
right parsopercularis	156.203	1.670	**	-499.921	180.338	**	25.403	2.496	**	541.736	264.245	n.s.
right parsorbitalis	174.711	1.870	**	50.267	201.936	n.s.	25.558	2.795	**	38.219	295.892	n.s.
right parstriangularis	77.382	0.794	**	363.966	85.693	**	7.429	1.186	**	-146.078	125.564	n.s.
right pericalcarine	184.815	1.887	**	262.275	203.816	n.s.	21.655	2.821	**	94.098	298.647	n.s.
right postcentral	184.562	1.820	**	86.236	196.575	n.s.	13.005	2.720	**	-583.514	288.038	n.s.
right posteriorcingulate	331.128	3.494	**	-256.202	377.343	n.s.	43.218	5.222	**	-322.962	552.913	n.s.
right precentral	133.855	1.413	**	118.978	152.606	n.s.	14.929	2.112	**	61.943	223.610	n.s.
right precuneus	373.987	4.130	**	254.919	446.051	n.s.	53.178	6.173	**	-1474.581	653.589	n.s.
right rostralanteriorcingulate	355.954	3.689	**	491.740	398.397	n.s.	42.230	5.513	**	-70.807	583.762	n.s.
right rostralmiddlefrontal	97.209	1.006	**	-322.561	108.606	**	10.462	1.503	**	194.938	159.138	n.s.
right superiorfrontal	562.107	5.703	**	1950.479	615.924	**	62.001	8.524	**	-562.373	902.501	n.s.
right superiorparietal	586.732	6.069	**	1396.682	655.391	*	73.254	9.070	**	99.951	960.330	n.s.
right superiortemporal	453.249	4.721	**	768.519	509.852	n.s.	49.896	7.056	**	-407.728	747.076	n.s.
right supramarginal	281.280	2.903	**	608.371	313.474	n.s.	31.507	4.338	**	-694.847	459.327	n.s.
right frontalpole	375.950	3.841	**	732.416	414.824	n.s.	52.182	5.741	**	-29.682	607.832	n.s.
right temporalpole	34.365	0.353	**	-16.406	38.079	n.s.	2.976	0.527	**	48.350	55.796	n.s.
right transversetemporal	44.135	0.457	**	150.427	49.340	**	5.188	0.683	**	10.036	72.297	n.s.
right insula	43.318	0.436	**	-38.027	47.047	n.s.	4.407	0.651	**	134.689	68.938	n.s.

Supplementary Table 3C

Thickness	Intercept	(s.e.)	P	Age2	(s.e.)	P	Sex	(s.e.)	P	Sex by age2	(s.e.)	P
left bankssts	0.084	0.001	**	2.320	0.095	**	0.005	0.001	**	0.066	0.135	n.s.
left caudalanteriorcingulate	0.141	0.001	**	1.885	0.156	**	0.003	0.002	n.s.	-0.247	0.222	n.s.
left caudalmiddlefrontal	0.206	0.002	**	2.014	0.223	**	-0.005	0.003	n.s.	0.158	0.317	n.s.
left cuneus	0.124	0.001	**	1.968	0.140	**	0.005	0.002	**	0.366	0.198	n.s.
left entorhinal	0.111	0.001	**	1.243	0.125	**	0.003	0.002	n.s.	0.300	0.177	n.s.
left fusiform	0.266	0.003	**	1.105	0.298	**	0.002	0.004	n.s.	0.744	0.424	n.s.
left inferiorparietal	0.116	0.001	**	1.049	0.131	**	0.002	0.002	n.s.	0.428	0.186	n.s.
left inferiortemporal	0.112	0.001	**	2.475	0.126	**	0.006	0.002	**	-0.005	0.178	n.s.
left isthmuscingulate	0.131	0.001	**	1.354	0.145	**	0.002	0.002	n.s.	0.112	0.205	n.s.
left lateraloccipital	0.168	0.002	**	2.249	0.181	**	-0.002	0.002	n.s.	-0.062	0.257	n.s.
left lateralorbitofrontal	0.099	0.001	**	1.086	0.111	**	0.005	0.001	**	0.336	0.158	n.s.
left lingual	0.130	0.001	**	2.192	0.148	**	0.008	0.002	**	0.370	0.211	n.s.
left medialorbitofrontal	0.102	0.001	**	1.786	0.115	**	0.002	0.001	n.s.	-0.019	0.163	n.s.
left middletemporal	0.140	0.001	**	2.002	0.158	**	0.002	0.002	n.s.	-0.220	0.224	n.s.
left parahippocampal	0.132	0.001	**	1.873	0.148	**	0.006	0.002	**	0.414	0.210	n.s.
left paracentral	0.249	0.002	**	0.884	0.263	**	0.004	0.003	n.s.	0.709	0.373	n.s.
left parsopercularis	0.129	0.001	**	2.123	0.143	**	0.004	0.002	*	0.110	0.203	n.s.
left parsorbitalis	0.127	0.001	**	2.030	0.139	**	0.006	0.002	**	-0.150	0.198	n.s.
left parstriangularis	0.183	0.002	**	2.713	0.200	**	0.006	0.003	*	-0.017	0.284	n.s.
left pericalcarine	0.138	0.001	**	2.138	0.152	**	0.007	0.002	**	0.439	0.216	n.s.
left postcentral	0.104	0.001	**	0.810	0.121	**	0.001	0.002	n.s.	0.196	0.171	n.s.
left posteriorcingulate	0.099	0.001	**	1.541	0.111	**	0.005	0.001	**	0.312	0.157	n.s.
left precentral	0.134	0.001	**	2.195	0.147	**	0.004	0.002	*	-0.051	0.209	n.s.
left precuneus	0.113	0.001	**	1.752	0.126	**	0.004	0.002	**	-0.019	0.179	n.s.
left rostralanteriorcingulate	0.113	0.001	**	2.472	0.125	**	0.004	0.002	**	0.354	0.178	n.s.
left rostralmiddlefrontal	0.195	0.002	**	2.076	0.213	**	-0.004	0.003	n.s.	0.088	0.302	n.s.
left superiorfrontal	0.115	0.001	**	2.092	0.131	**	0.007	0.002	**	0.617	0.186	*
left superiorparietal	0.127	0.001	**	2.679	0.142	**	0.003	0.002	n.s.	0.265	0.202	n.s.
left superiortemporal	0.101	0.001	**	2.096	0.113	**	0.005	0.001	**	-0.027	0.160	n.s.
left supramarginal	0.131	0.001	**	2.277	0.143	**	0.005	0.002	**	0.090	0.203	n.s.
left frontalpole	0.119	0.001	**	2.386	0.129	**	0.005	0.002	**	-0.027	0.184	n.s.
left temporalpole	0.248	0.002	**	2.941	0.275	**	0.005	0.003	n.s.	0.335	0.391	n.s.
left transversetemporal	0.267	0.003	**	0.298	0.306	n.s.	0.006	0.004	n.s.	1.223	0.434	n.s.
left insula	0.184	0.002	**	2.050	0.200	**	0.000	0.003	n.s.	0.296	0.284	n.s.
right bankssts	0.128	0.001	**	1.806	0.142	**	0.003	0.002	n.s.	0.212	0.201	n.s.
right caudalanteriorcingulate	0.149	0.001	**	2.170	0.163	**	0.004	0.002	n.s.	-0.116	0.231	n.s.
right caudalmiddlefrontal	0.188	0.002	**	2.080	0.206	**	-0.005	0.003	*	-0.227	0.292	n.s.
right cuneus	0.124	0.001	**	1.943	0.136	**	0.003	0.002	n.s.	-0.065	0.193	n.s.
right entorhinal	0.113	0.001	**	1.416	0.124	**	0.002	0.002	n.s.	0.045	0.177	n.s.
right fusiform	0.290	0.003	**	1.469	0.318	**	0.003	0.004	n.s.	0.460	0.452	n.s.
right inferiorparietal	0.116	0.001	**	1.602	0.131	**	0.002	0.002	n.s.	0.178	0.186	n.s.
right inferiortemporal	0.113	0.001	**	2.722	0.127	**	0.007	0.002	**	0.056	0.180	n.s.
right isthmuscingulate	0.128	0.001	**	1.380	0.144	**	0.004	0.002	*	0.281	0.205	n.s.
right lateraloccipital	0.164	0.002	**	2.094	0.177	**	-0.001	0.002	n.s.	-0.040	0.252	n.s.
right lateralorbitofrontal	0.104	0.001	**	1.331	0.116	**	0.007	0.001	**	0.599	0.164	*
right lingual	0.135	0.001	**	2.002	0.153	**	0.005	0.002	**	0.034	0.218	n.s.
right medialorbitofrontal	0.105	0.001	**	1.756	0.117	**	0.001	0.001	n.s.	0.186	0.167	n.s.
right middletemporal	0.148	0.001	**	1.745	0.167	**	0.005	0.002	*	0.194	0.236	n.s.
right parahippocampal	0.127	0.001	**	1.952	0.142	**	0.006	0.002	**	0.103	0.202	n.s.
right paracentral	0.208	0.002	**	1.224	0.230	**	0.005	0.003	n.s.	-0.332	0.326	n.s.
right parsopercularis	0.126	0.001	**	1.900	0.139	**	0.004	0.002	*	0.355	0.197	n.s.
right parsorbitalis	0.134	0.001	**	1.732	0.145	**	0.002	0.002	n.s.	-0.161	0.205	n.s.
right parstriangularis	0.179	0.002	**	2.199	0.196	**	0.004	0.002	n.s.	0.389	0.278	n.s.
right pericalcarine	0.135	0.001	**	1.844	0.147	**	0.001	0.002	n.s.	-0.122	0.208	n.s.
right postcentral	0.105	0.001	**	0.793	0.120	**	0.002	0.002	n.s.	0.050	0.171	n.s.
right posteriorcingulate	0.105	0.001	**	1.942	0.118	**	0.001	0.001	n.s.	0.118	0.167	n.s.
right precentral	0.133	0.001	**	2.117	0.146	**	0.000	0.002	n.s.	0.109	0.207	n.s.
right precuneus	0.113	0.001	**	1.694	0.127	**	0.005	0.002	**	0.133	0.181	n.s.
right rostralanteriorcingulate	0.113	0.001	**	2.499	0.125	**	0.005	0.002	**	0.244	0.178	n.s.
right rostralmiddlefrontal	0.188	0.002	**	1.992	0.212	**	0.008	0.003	**	-0.059	0.301	n.s.
right superiorfrontal	0.113	0.001	**	1.736	0.130	**	0.005	0.002	**	0.250	0.184	n.s.
right superiorparietal	0.125	0.001	**	2.589	0.139	**	0.004	0.002	*	0.117	0.197	n.s.
right superiortemporal	0.103	0.001	**	1.986	0.115	**	0.004	0.001	**	0.361	0.164	n.s.
right supramarginal	0.128	0.001	**	2.116	0.141	**	0.007	0.002	**	0.191	0.201	n.s.
right frontalpole	0.121	0.001	**	2.782	0.133	**	0.005	0.002	**	0.020	0.189	n.s.
right temporalpole	0.243	0.002	**	2.417	0.269	**	0.003	0.003	n.s.	0.592	0.382	n.s.
right transversetemporal	0.274	0.003	**	0.825	0.323	*	0.003	0.004	n.s.	1.082	0.459	n.s.
right insula	0.183	0.002	**	1.833	0.203	**	0.010	0.003	**	0.211	0.288	n.s.
